# Performance evaluation and multi-objective optimization of protective slurry seal asphalt mixtures incorporating foundry waste filler and silver nanoparticles

**DOI:** 10.1038/s41598-025-23275-6

**Published:** 2025-11-12

**Authors:** Vahid Bagheri Mofrad, Mahmood Jazayeri Moghaddas, Amin Tohidi, Hassan Divandari, Mehdi Mahdavi Adeli

**Affiliations:** 1 Department of Mining Engineering, Amirkabir Universit of Technology, Tehran, Iran; 2 Department of Civil Engineering, CT.C., Islamic Azad University, Tehran, Iran; 3 Department of Civil Engineering, Shoushtar Branch, Islamic Azad University, Shoushtar, Iran; 4 Department of Civil Engineering, WT.C., Islamic Azad University, Tehran, Iran

**Keywords:** Protective asphalt, Slurry seal, Foundry waste filler, Silver nanoparticles, Multi-objective modeling, Engineering, Environmental sciences, Materials science

## Abstract

This study presents a comprehensive evaluation of protective slurry seal (SS) asphalt mixtures incorporating foundry waste filler (FWF) and silver nanoparticles (AgNPs). The novelty of this research lies in the combined use of an industrial by-product (FWF) with nanomaterials (AgNPs), a concept that has not been previously reported in SS technology. The main objective is to improve the sustainability and mechanical behavior of SSs by partially replacing natural fillers with FWF and enhancing the microstructural properties through AgNPs. To achieve this, an extensive experimental program was conducted, including chemical characterization (XRF, XRD, FTIR), microstructural analysis (FESEM), and rheological as well as mechanical tests such as wet track abrasion (WTAT), loaded wheel test (LWT), and sand adhesion. The results revealed that FWF significantly improved filler packing and bonding capacity, while the addition of AgNPs enhanced adhesion and rutting resistance. The combined use of FWF and AgNPs produced superior durability compared to the control mixtures. Among the tested samples, the mixture containing 75% FWF and 10% AgNPs provided the most balanced performance, showing improvements of up to 20–25% in adhesion and rutting resistance relative to the reference. A multi-objective optimization approach confirmed this composition as the optimal solution, considering both technical performance and sustainability indicators. These findings also demonstrate practical implications, including reduced material costs, lower environmental footprint, and potential feasibility for large-scale field applications in pavement engineering.

## Introduction

In recent years, colored asphalt mixtures have been increasingly used in urban areas for safety and aesthetic purposes, such as pedestrian crossings, traffic islands, and dedicated lanes. Although pigments influence durability and mixture design, their role is limited compared to sustainable additives and nanotechnology. Therefore, in this paper, pigment-related aspects are briefly mentioned only as contextual background, while the focus is placed on nanoparticle-modified slurry seals (SS) incorporating industrial by-products^1^.

In recent years, several studies have sought to improve the performance of SS. In one such study, Poursoltani and Hesami (2018) investigated the use of various amounts of reclaimed asphalt pavement (RAP) in SS mixtures. Suitable mix designs were prepared for samples containing 43%, 69%, and 95% RAP, as well as a control mix with 100% virgin aggregate. The initial design tests showed that while RAP mixtures met standard specifications, they required slightly more bitumen than virgin mixtures to achieve adequate cohesion within the designated timeframe. Among the RAP-based mixes, the one with 69% RAP exhibited the best performance^2^. In another study, Zalnezhad and Hesami (2020) assessed the effect of using steel slag on the performance of SS. The control mix contained 100% siliceous aggregate, and the slag replaced the aggregate at rates of 42.5%, 61%, and 100%. Performance was evaluated using wet cohesion tests (at 30 and 60 min), wet abrasion tests, and vertical/lateral displacement under a loaded wheel. The results showed that steel slag improved the mechanical, physical, and chemical properties of the mixtures. The mix containing 61% slag demonstrated the least displacement, reducing vertical and lateral deformation by 45.65% and 35%, respectively, compared to the control^3^.

In another study, Otadi and Tanzadeh (2018) evaluated SS mixtures modified with nanomaterials and polyethylene fibers. The additives included 4% nano-silica and 3% nano-clay (based on residual bitumen weight), which were added to the bitumen emulsion. In addition, 4% polyethylene fibers (based on dry aggregate weight) were incorporated into the asphalt mix. The appropriate mix design was determined using loaded wheel, wet abrasion, and wet cohesion tests. Results showed that the addition of nano-silica after emulsion breaking increased cohesion by 8% for the emulsion and 5% for the mix. Nano-clay enhanced abrasion resistance by 12%, while fiber addition reduced displacement by 27%^4^.

Despite these advances, there remains a clear research gap: limited studies have investigated sustainable filler alternatives in SS mixtures that are simultaneously enhanced with nanomaterials. In particular, the combined use of foundry waste filler (FWF) and silver nanoparticles (AgNPs) in protective SSs has not yet been systematically studied.

### Research significance and objectives

The significance of this study lies in addressing both sustainability and performance in SSs by integrating industrial by-products with nanotechnology. The objectives are: (i) to evaluate the effect of partially or fully replacing natural fillers with foundry waste fillers, (ii) to assess the influence of AgNPs on mechanical and durability performance, (iii) to develop predictive models for performance indicators, and (iv) to identify the optimal mixture through multi-objective optimization. By bridging the gap in previous studies, this research provides both technical insights and practical implications for sustainable pavement engineering.

## Materials

A standard SS mixture is composed of fully crushed aggregate with controlled gradation, polymer-modified cationic bitumen emulsion, water, mineral filler, and, when required, chemical additives to regulate the emulsion’s breaking time. Each of these components must comply with the specifications set forth in ISSA A143, as outlined in relevant standards^5,6^.

### Aggregates

The aggregates used in this study serve as the primary skeleton of the protective asphalt mix, playing a critical role in the mechanical strength, surface durability, and overall performance behavior of the mixture. Crushed aggregates with a rough texture were selected to enhance contact with the bitumen emulsion, ensuring improved adhesion and sufficient stability under loading conditions. According to the ISSA TB-111 standard for SS design, aggregates should primarily fall within the fine size range (passing sieve No. 8). The final gradation distribution of the aggregates is presented in Table 1. In this study, the fraction passing the 0.075 mm sieve—referred to as filler—was composed of natural and/or waste fillers combined with nanomaterials and will be discussed in a separate section. The physical properties of the aggregates were determined after sampling and laboratory analysis and are summarized in Table 2.

**Table 1:** Gradation of aggregates for SS mixture.

Sieve size	Type II used (% passing)	ISSA type II specification range (%)	Permissible tolerance relative (%)
4.75 mm (No. 4)	100	90–100	± 5
2.36 mm (No. 8)	77.5	65–90	± 5
1.18 mm (No. 16)	57.5	45–70	± 5
600 μm (No. 30)	40	30–50	± 5
300 μm (No. 50)	24	18–30	± 5
150 μm (No. 100)	15.5	10–21	± 4
75 μm (No. 200)	10	5–15	± 3

**Table 2:** Physical properties of aggregates used in the SS mixture.

Property	Measured value	Unit / Reference
Bulk specific gravity	2.58	g/cm^3^
Saturated surface-dry (SSD) specific gravity	2.61	g/cm^3^
Apparent specific gravity	2.65	g/cm^3^
Water absorption	1.2	%
Fractured face percentage	> 95	% (ISSA TB-112)
Clay or harmful fine particles content	< 1	%

### Polymer-modified Bitumen emulsion

The bitumen emulsion utilized in this study was a cationic slow-setting type (CSS-1 h), chosen for its strong compatibility with SS and cold-mix applications. This emulsion offers excellent dispersion characteristics and performs effectively under ambient temperature conditions^7^. Its use also supports the project’s environmental goals, as it eliminates the need for high-temperature processing, thereby reducing emissions of harmful gases.

The bitumen emulsion was evaluated based on its physical and chemical properties, including residue content, penetration grade, softening point, and FT-IR spectrum. These characteristics were essential for assessing the emulsion’s suitability for blending with various fillers and nanomaterial additives^8^. Table 3 presents the key properties of the bitumen emulsion according to standard test methods.

**Table 3:** Properties of the Bitumen emulsion used in this study.

Test	Test method	Test result
Saybolt furol viscosity at 25 °C (seconds)	AASHTO T59	21
Storage stability of bitumen emulsion (24 h, %)	ASTM D6930	0.1
Distillation residue of emulsion (by weight, %)	ASTM D7497	62
Sieve test (%)	ASTM D244	0.1
Coating ability (%)	AASHTO T59	99
Particle charge	AASHTO T59	Positive

### Natural filler

The natural filler used in this study was limestone powder of carbonate origin, produced industrially by grinding stone aggregates. This type of filler is widely used in protective asphalt and fine aggregate industries due to its availability, low cost, and compatibility with bitumen emulsions. The primary purpose of using this filler as a baseline in the present study was to evaluate the performance of alternative fillers and their behavior in the presence of nanomaterials.

To this end, the natural filler was evaluated from physical, chemical, and structural perspectives. Structural analysis using X-ray diffraction (XRD) revealed that the dominant phase in the filler is calcite (CaCO₃). Additionally, the diffraction spectrum exhibited distinct peaks related to dolomite, indicating the presence of minor quantities of this mineral. For a more detailed understanding of molecular structure and surface functional groups, FT-IR spectroscopy was performed. The recorded spectrum displayed characteristic peaks at:

- 1420–1470 cm^−1^: stretching vibrations of carbonate (CO₃²⁻) groups.
- 875 cm^−1^: out-of-plane bending of carbonate.
- 710 cm^−1^: in-plane bending of carbonate.
- ~ 3400 cm^−1^: surface –OH groups due to moisture adsorption.

These spectral features confirm the predominance of calcium carbonate and the inorganic mineral nature of the filler.

Overall, although the natural filler exhibited acceptable performance, it showed inferior results in certain parameters such as abrasion resistance and displacement when compared with nano-modified waste filler. As the reference material in this study, natural filler provided a suitable basis for evaluating the influence of industrial waste and nanoparticles on the performance of protective asphalt mixtures^9^.

### Foundry waste filler

The waste filler used in this study was derived from CO₂ molding sand residues from the metal casting process^10^. These residues, typically considered waste in the metal industry, can be processed and reused as a sustainable substitute for natural filler in protective asphalt mixtures. Using this type of filler not only reduces costs and conserves natural resources but also promotes environmental sustainability in road construction^11^.

After collection from foundries, the waste filler was dried, ground, and sieved through a 0.075 mm mesh to match the gradation of natural filler. This material exhibited a light gray color, coarser surface texture, and less uniform gradation compared to carbonate-based filler^12^. The primary chemical components of the waste filler included:

- SiO_2_ (Silicon Dioxide): in high concentration (> 60%).
- Al_2_O_3_ and Fe_2_O_3_: in significant amounts.
- Minor contents of CaO, MgO, and other metallic oxides.

This composition indicates a siliceous and quasi-ceramic nature, contrasting with the alkaline properties of natural filler^13^.

Moreover, secondary minerals such as mullite and traces of kaolinite were observed in some samples. The FT-IR spectrum of the waste filler showed the following characteristic peaks:

- ~ 1080 cm^−1^: Si–O–Si stretching vibrations.
- ~ 795 cm^−1^ and ~ 470 cm^−1^: Si–O bending vibrations.
- ~ 1630 cm^−1^ and ~ 3400 cm^−1^: moisture adsorption and surface –OH groups.

These findings confirm that the molecular composition of the filler belongs to the silicate family and lacks active carbonate groups, unlike the natural filler.

Given its siliceous nature, the adhesion behavior of this filler with bitumen is expected to differ. Mastic test results indicated that in the absence of nanomaterials, increasing the proportion of waste fillers could initially reduce adhesion. However, in the presence of nanomaterials, this effect improved, and the waste filler demonstrated favorable performance. In some samples, such as NWFP50, significant improvements in abrasion resistance and reduction in deformation were observed.

Overall, foundry waste filler—with its silicate structure, suitable gradation, and wide availability—emerges as a viable and sustainable alternative to traditional fillers. When structurally enhanced through nanomaterials, it can contribute to improved mechanical properties and durability in protective asphalt mixtures.

### Nanomaterials (type, dosage, and mixing method)

To enhance the performance of protective asphalt mixtures and improve the mechanical and rheological properties of bituminous mastics, silver nanoparticles (AgNPss) were employed in this study as a modifying additive. The physical and chemical properties of AgNPss are presented in Table 4. The use of metallic nanoparticles in bituminous mixes—owing to their high surface activity, ultrafine particle size, and unique physicochemical properties—can enhance adhesion behavior, increase resistance to abrasion and deformation, and reduce temperature sensitivity.

The AgNPs used in this research possessed the following characteristics:

- High thermal and electrical conductivity.
- Ability to interact physically and chemically with the bitumen matrix.
- High stability at intermediate and elevated temperatures.
- Proven effectiveness in improving tensile strength and mix cohesion.

**Table 4:** Physical and chemical properties of silver nanoparticles.

Property	Approximate value	Unit
Average particle size	40–70	nm
Density	~ 8.9	g/cm^3^
Purity	> 99	wt%
Specific surface area (BET)	~ 50–80	m^2^/g
Morphology	Spherical and crystalline	Dimensionless

## Mix design and modeling

In this study, the optimal dosage of AgNPs was determined to be 10% by weight relative to the total filler content. This value was selected to ensure both adequate processability during mixing and a significant effect on enhancing the mechanical and rheological behavior of the bituminous mastic. In other words, in samples containing nanomaterials (e.g., NWFP50), 10% of the total filler weight (including both natural and waste fillers) was replaced with AgNPs.

To achieve a uniform dispersion of AgNPs within the mixture, the mixing procedure was conducted as follows:

1. Pre-mixing of AgNPs with dry filler in a high-speed mixer for 3 min.
2. Addition of the pre-mixed material to the bitumen emulsion at a temperature of 60–70 °C.
3. Stirring the blend using a magnetic hotplate stirrer for 10 min to facilitate the uniform distribution of nanoparticles within the bitumen matrix.

This method ensured a homogeneous distribution of AgNPs throughout the mastic, improving the mixture’s cohesion and structural stability.

**Fig. 1:**
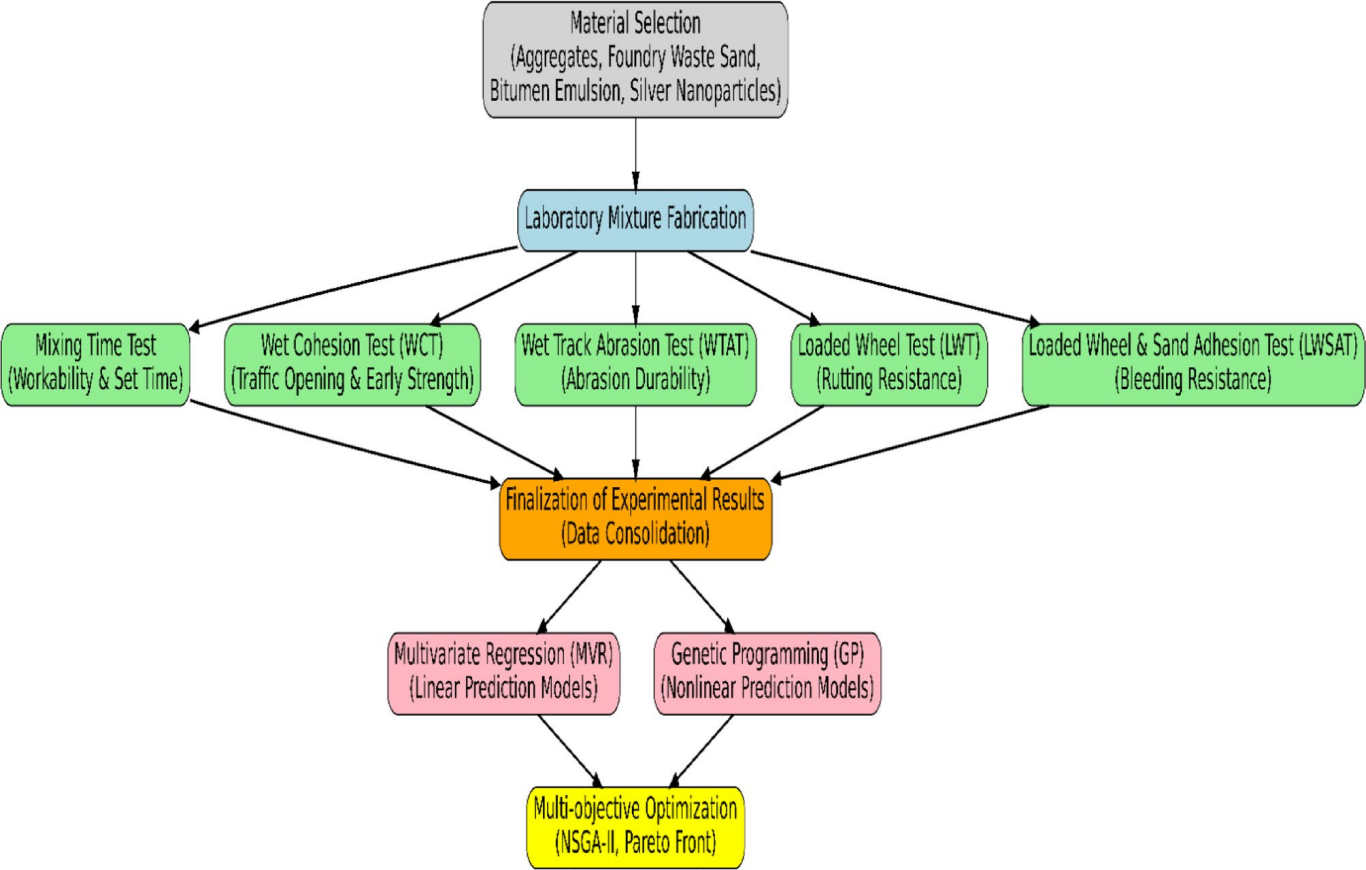
Steps of the present study for the design and performance evaluation of protective SS.

For clarity, all mixture types and their corresponding codes are summarized in Table 5. This table provides a concise reference to the combinations of natural filler, foundry waste filler, and AgNPs used throughout this study. To organize the research process coherently and ensure comprehensive coverage of the study objectives, the flowchart presented in Fig. 1 was developed. This chart outlines the various stages of the research, from material selection to final data analysis and extraction of the optimal mixture design, in a step-by-step logical framework.

Step 1: Mix Design Formulation: Based on the study’s objectives, experimental mixtures were designed using five levels of filler replacement (ranging from 0% to 100%) and three levels of residual bitumen content (8%, 8.5%, and 9%). These mixtures were categorized into two main groups: those with nanomaterials and those without, enabling comparative performance evaluation.

Step 2: Sample Classification: At this stage, the samples were classified according to the presence or absence of nanomaterials. Each mix design had two versions: one incorporating nanomaterials (such as silver nanoparticles) and one without. This dual structure allowed for the direct investigation of the impact of nanoparticles on mastic performance.

Step 3: Sample Preparation and Curing: Samples were fabricated according to the specified mix design procedure and cured under controlled conditions (temperature, humidity, mixing time, and molding). The optimal mixing time for each blend was determined using the Mixing Time Test.

Step 4: Laboratory Testing: A set of laboratory tests was conducted in alignment with the research objectives to evaluate various behavioral and performance characteristics of the mixtures.

Step 5: Statistical Analysis and Modeling: The experimental results were analyzed using two modeling approaches:

- Multivariate regression, to establish statistical relationships between input and output variables.
- Genetic programming (NSGA-II), to predict performance behavior and optimize mix compositions.

Final Step: Interpretation and Optimization: Finally, the results were interpreted to determine the optimal residual bitumen content and the most suitable levels of waste filler and nanomaterials needed to achieve superior mechanical performance and durability.

**Table 5:** Mixture types and codes.

Mixture type	Waste filler replacement (%)	Nano content (%)	Notes
WFP0	Natural filler only	–	Control (no nano)
WFP0	Natural filler only	–	Control (no nano)
WFP0	Natural filler only	–	Control (no nano)
NWFP0	Natural filler only	10	Nano-modified
NWFP0	Natural filler only	10	Nano-modified
NWFP0	Natural filler only	10	Nano-modified
NWFP25	25% waste filler	10	Nano-modified
NWFP25	25% waste filler	10	Nano-modified
NWFP25	25% waste filler	10	Nano-modified
NWFP50	50% waste filler	10	Nano-modified
NWFP50	50% waste filler	10	Nano-modified
NWFP50	50% waste filler	10	Nano-modified
NWFP75	75% waste filler	10	Nano-modified
NWFP75	75% waste filler	10	Nano-modified
NWFP75	75% waste filler	10	Nano-modified
NWFP100	100% waste filler	10	Nano-modified
NWFP100	100% waste filler	10	Nano-modified
NWFP100	100% waste filler	10	Nano-modified

### Labaratory test

#### Mixing time test

The mixing time test is a fundamental procedure in the design of SS mixtures, serving to determine suitable material proportions and assess mix workability. This test entails combining predetermined ratios of aggregate, filler, water, and bitumen emulsion^14^. The general composition limits for SS formulations are provided in Table 6.

To perform the test, 200 g of the selected aggregate—dried to a moisture content below 1%—is blended with varying amounts of mineral filler. Water and bitumen emulsion are then added to the dry mix, and the materials are stirred for 30 s at a temperature of 25 °C. Following this initial mixing phase, half of the mixture is spread onto a sheet of paper for visual assessment, while the remaining half is continuously stirred until the mix becomes noticeably stiff and the emulsion breaks. The elapsed time from the start of mixing to this point is recorded as the mixing time. Concurrently, the portion spread on the paper is intermittently touched with a fingertip; the time at which the surface resists deformation under finger pressure is noted as the set time^14^.

**Table 6:** Recommended ranges for SS components^6^.

Component	Recommended range
Residual Bitumen	5.5% to 10.5% by weight of dry aggregate
Mineral filler	0% to 3% by weight of dry aggregate
Polymer content	3% based on bitumen weight
Water	As required to achieve suitable consistency in the mixture

#### Wet cohesion test (WCT)

The wet cohesion test (WCT), conducted in accordance with ISSA TB 139^15^, is designed to assess the early-stage cohesion, traffic resistance, and curing behavior of SS mixtures. The curing process is considered complete once sufficient bonding between aggregate particles is achieved. A cohesion testing apparatus is employed to determine the time required to reach adequate cohesion. For a mixture to be deemed acceptable, it must meet the minimum cohesion thresholds of 12 kg·cm after 30 min and 20 kg·cm after 60 min of curing at room temperature (25 °C). The 30-minute measurement reflects the emulsion’s breakage characteristics, while the 60-minute value is indicative of traffic-readiness.

During the test, the slurry mixture is placed into ring molds (10 mm in height and 60 mm in diameter) suitable for Type II gradation, and the molds are set atop felt pads at ambient conditions. After sufficient setting, the molds are removed and the samples are tested using a cohesion device that applies a torsional load. The pressure exerted by the cylinder on the sample is maintained at 200 kPa. The resulting cohesion-time curve indicates whether the mixture sets rapidly or slowly, thereby determining its suitability for either high-speed or low-speed traffic environments^15^.

#### Wet track abrasion test (WTAT)

The Wet Track Abrasion Test (WTAT) is employed to evaluate the raveling resistance of SS mixtures, assess the quality of aggregate coating under wet, abrasive conditions, and identify the minimum bitumen emulsion content necessary to prevent raveling-related failures. This test simulates surface abrasion, stripping, and traffic-induced wear—such as braking and turning—in the presence of water. It quantifies the mass loss of aggregates subjected to wet abrasion using a rotating rubber hose.

**Fig. 2:**
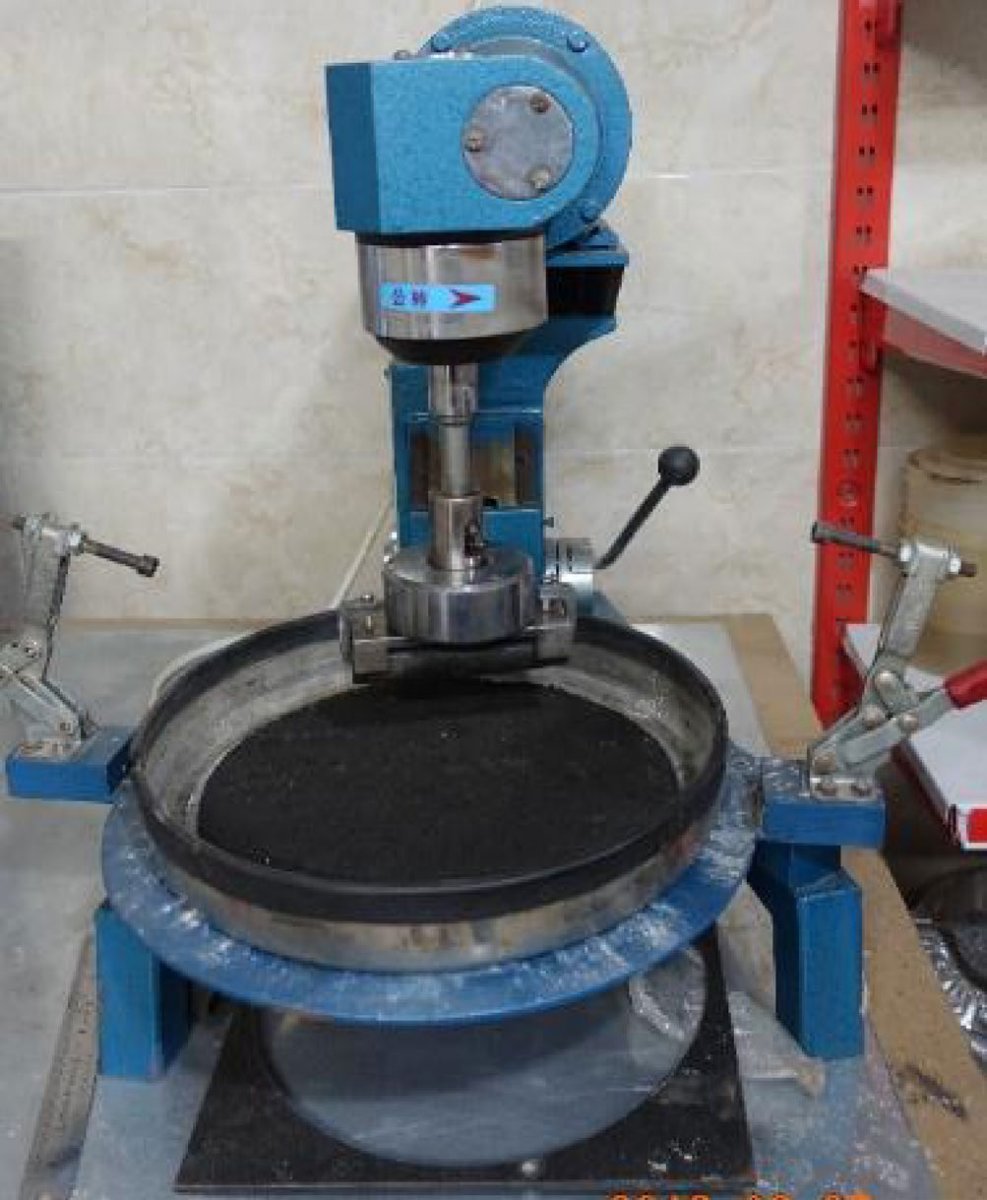
View of the wet track abrasion test (WTAT) apparatus.

In this procedure, a cured SS specimen with a thickness of 6 mm and a diameter of 280 mm is submerged in water at 25 °C for six days. Following this conditioning period, the specimen is abraded with a 2.3 kg rubber hose rotating on its surface for five minutes while still underwater. The sample is then dried at 60 °C and weighed to determine the mass loss^16^. A schematic representation of the test apparatus is provided in Fig. 2. According to ISSA A143 standards, the maximum permissible mass loss for SS mixtures over six days of wet abrasion is 807 g/m².

#### Loaded wheel & sand adhesion test (LWSAT)

**Fig. 3:**
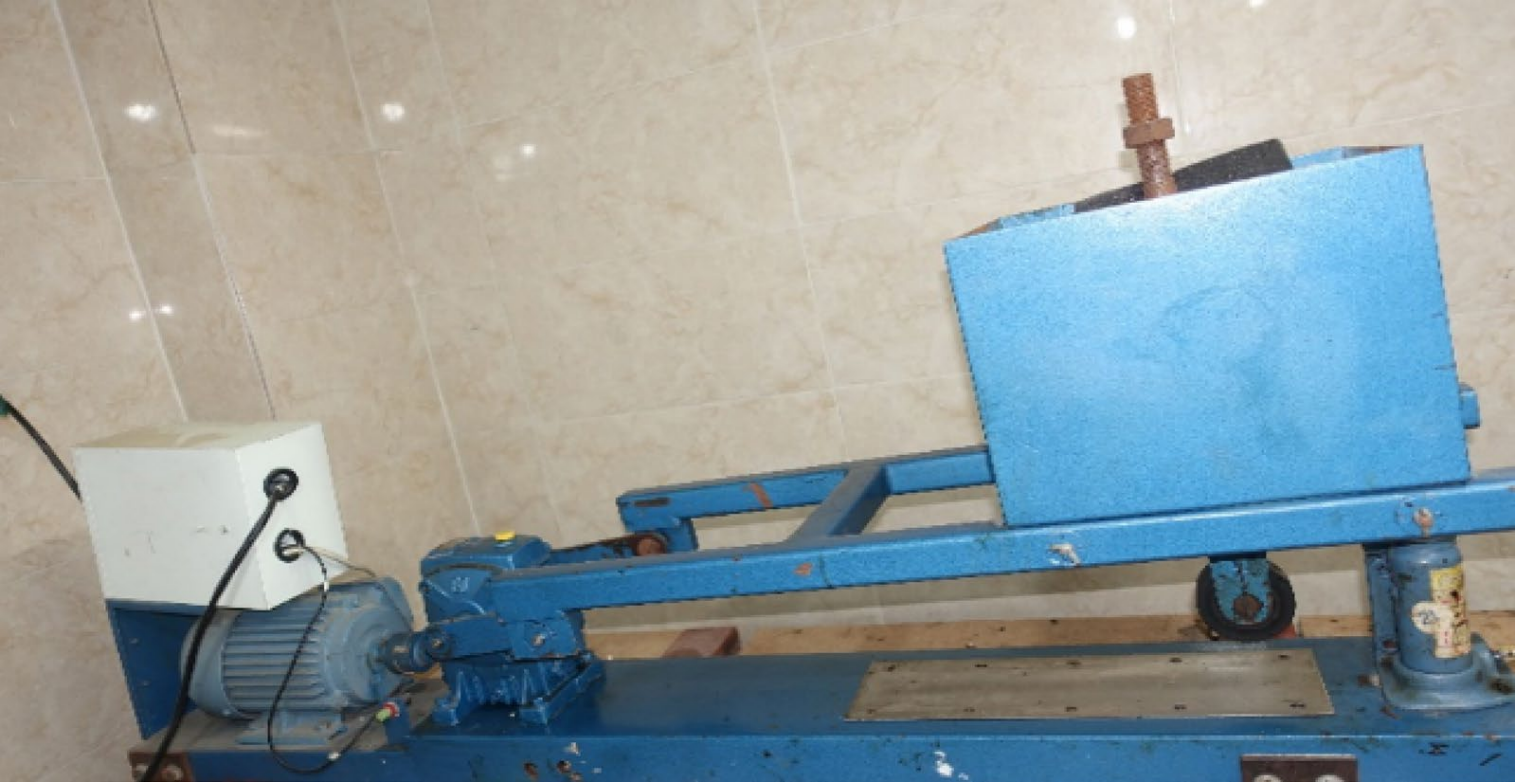
Loaded wheel tester apparatus.

- The Loaded Wheel and Sand Adhesion Test (LWSAT) is conducted to determine the minimum bitumen content necessary to prevent surface defects such as bleeding or flushing in SS mixtures. In this procedure, the mixture is first compacted under a loaded wheel weighing 56.7 kg for 1000 cycles, after which the specimen is weighed and the initial mass is recorded.
- Subsequently, 200 g of Ottawa sand—graded according to specifications and preheated along with a metal strip to 82 °C—is uniformly distributed across the compacted surface. The same compaction process is then repeated for an additional 100 cycles to simulate continued traffic loading.
- Following the second compaction phase, the specimen is carefully removed, and any loose sand is gently brushed off. The specimen is then reweighed, and the difference in mass between the post-sand and pre-sand phases (after the initial 1000 cycles) represents the amount of sand retained on the surface^17^. This retained sand mass serves as an indirect measure of the bitumen content by evaluating the mixture’s ability to hold sand under simulated traffic conditions^16^. The test setup is illustrated in Fig. 3.

#### Loaded wheel test (LWT)

**Fig. 4:**
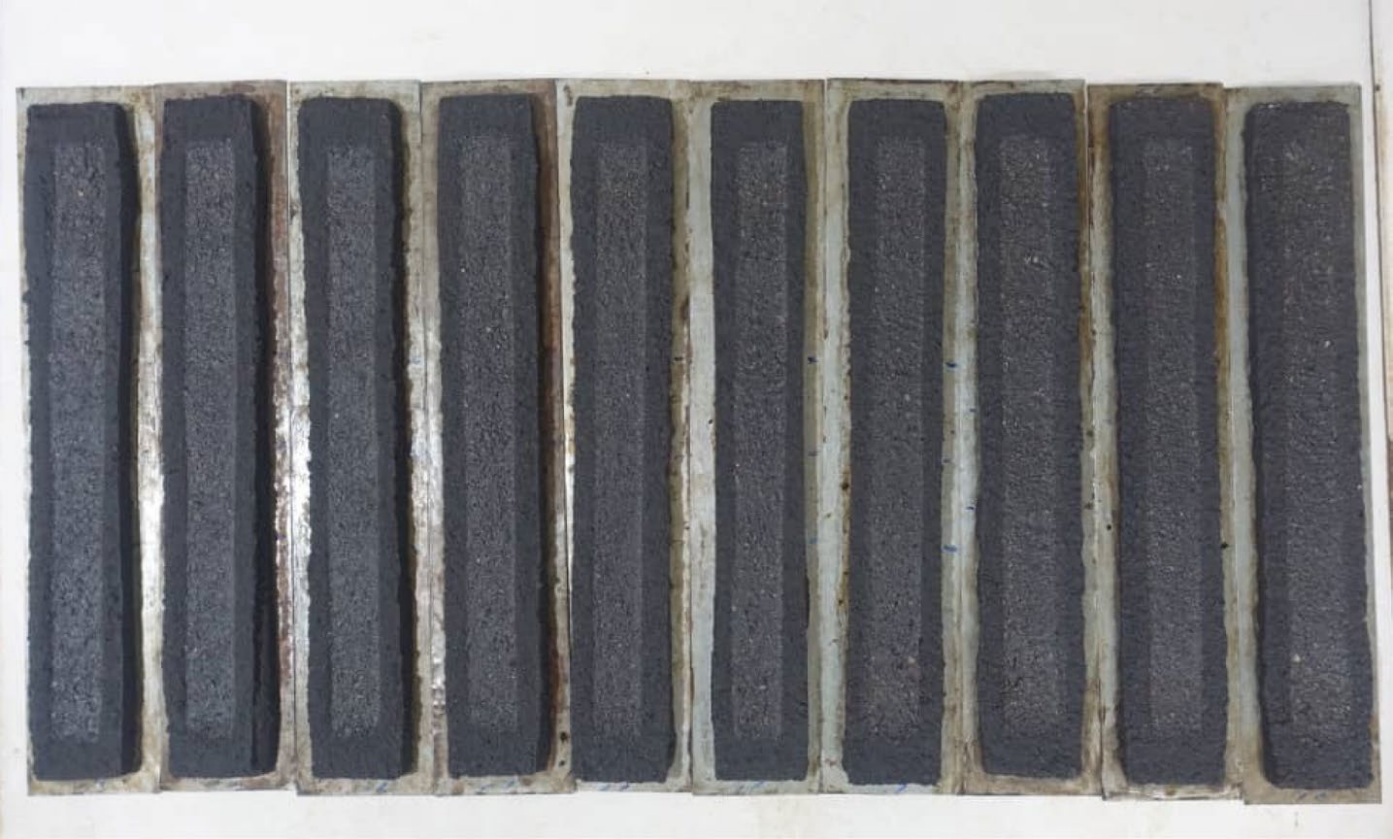
Specimens prepared for the loaded wheel test (LWT).

In this study, the LWT followed the shared compaction parameters defined in Sect. 3.1.4, with deformation recorded at the wheel path and mid-length positions. According to ISSA guidelines, acceptance criteria were ≤ 10% vertical deformation and ≤ 5% lateral displacement^18^. This cross-reference eliminates repetition of identical compaction details while preserving test-specific measurements and criteria. Figure 4 illustrates the test specimens after the completion of the loaded wheel displacement test.

### Modeling

#### Multivariate regression (MVR)

The Multiple Variable Regression (MVR) method utilizes a polynomial function to model the relationship between dependent and independent variables. To evaluate the model’s performance, statistical indicators such as the coefficient of determination (R²), adjusted R² (R_adj²), and root mean square error (RMSE) are employed. While R² quantifies the proportion of variance in the dependent variable explained by the independent variables, it can be misleading, as it tends to increase with the addition of more predictors and observations, potentially leading to overestimation. To address this limitation, the adjusted R² accounts for the number of predictors and adjusts the model accordingly, offering a more reliable measure of model fit and variable appropriateness.

In addition, the Pearson correlation coefficient (R) was used in this study to evaluate the strength and direction of linear relationships between quantitative variables. This coefficient ranges from + 1 (perfect positive correlation) to − 1 (perfect negative correlation), providing a straightforward measure of linear association^19^. Equations (1), (2), (3), and (4) provide the formulas for R, $\:{R}^{2}$, $\:{R}_{adj}^{2}$, and RMSE, respectively.

1 $$\:R=\frac{N\left(\sum\:xy\right)-\left(\sum\:x\right)\left(\sum\:y\right)}{\sqrt{\left[N\sum\:{x}^{2}-{\left(\sum\:x\right)}^{2}\right][N\sum\:{y}^{2}-{\left(\sum\:y\right)}^{2}]}}$$

2 $$\:{R}^{2}=\frac{{(\sum\:_{i=1}^{N}({y}_{actual}-{\stackrel{-}{y}}_{actual})\times\:({y}_{predicted}-{\stackrel{-}{y}}_{predicted}))}^{2}}{\sum\:_{i=1}^{N}{({y}_{actual}-{\stackrel{-}{y}}_{actual})}^{2}\times\:\sum\:_{i=1}^{N}{({y}_{predicted}-{\stackrel{-}{y}}_{predicted})}^{2}}$$

3 $$\:{R}_{adj}^{2}=1-\frac{\left(1-{R}^{2}\right)\left(N-1\right)}{N-P-1}$$

4 $$\:RMSE=\sqrt{\frac{\sum\:_{i=1}^{N}{({y}_{actual}-{\stackrel{-}{y}}_{predicted})}^{2}}{N}}$$

Where $\:x$ and $\:y$ are two variables, $\:N$ is number of the specimens, $\:p$ is number of predictor variables, $\:{y}_{actual}$ is laboratory value, $\:{\stackrel{-}{y}}_{actual}$ is mean laboratory value, $\:{y}_{predicted}$ is the value predicted, $\:{\stackrel{-}{y}}_{predicted}$ is the mean value predicted.

#### Genetic programming (GP)

Genetic programming (GP) is an advanced form of evolutionary algorithm inspired by Darwin’s theory of natural selection. Unlike traditional data-driven methods, GP explores the solution space directly to evolve models or functions that address a given problem. It operates as both an optimization and regression tool, generating solutions through iterative selection, crossover, and mutation—mimicking the process of natural evolution. A key advantage of GPis its ability to discover functional relationships without relying on predefined model structures. The final output is typically expressed as an explicit mathematical equation, offering interpretable results^20^.

Evolutionary algorithms such as NSGA-II are particularly effective for multi-objective optimization tasks, owing to their population-based search mechanisms. These algorithms overcome several limitations of classical approaches, for example, the need for repeated single-objective optimizations to construct a Pareto front or the use of weighted aggregations to balance competing objectives. By maintaining genetic diversity throughout the evolutionary process, these algorithms help prevent premature convergence and ensure a well-distributed set of solutions across the Pareto front^21^.

## Results and discussion

### Optimum residual Bitumen content

Determining the optimum residual bitumen content is a critical step in asphalt mixture design, as it directly influences key performance parameters such as adhesion, durability, abrasion resistance, and deformation behavior. In this study, the optimal bitumen content was identified based on the performance outcomes of two principal evaluation methods: the Wet Track Abrasion Test (WTAT) and the Loaded Wheel & Sand Adhesion Test (LWT-Displacement).

**Fig. 5:**
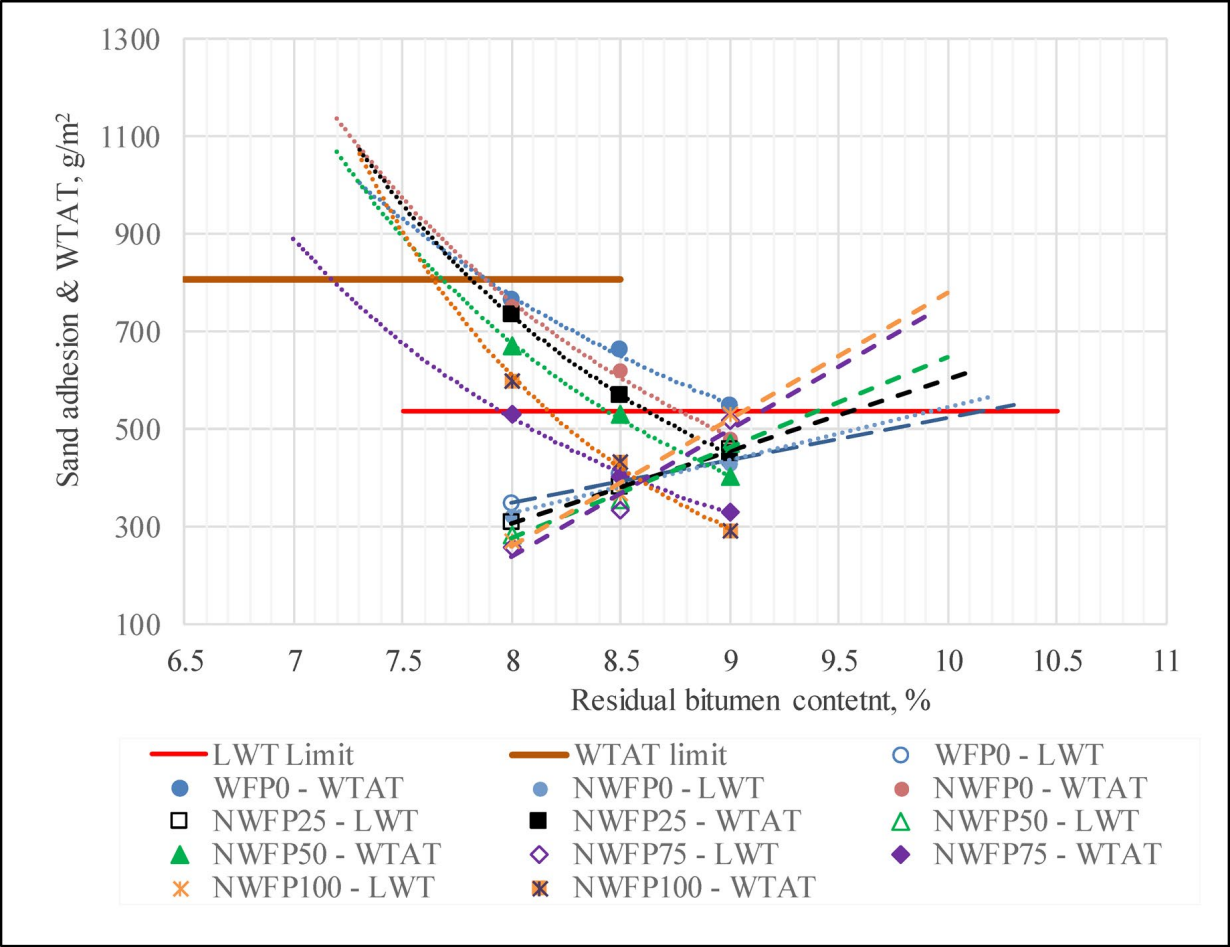
Overlapping graph of wet abrasion and wheel load & sand adhesion tests for determining optimum residual bitumen content.

For each combination of filler type and nanoparticle inclusion, samples were prepared and tested at three residual bitumen content levels: 8%, 8.5%, and 9%. Figure 5 presents an overlapping plot of the results from both tests across all experimental mixtures.

The optimum bitumen content was defined as the point at which:

- The values of abrasion loss and lateral displacement are minimized and remain within acceptable limits.
- Simultaneously, adhesion and cohesion performance remain at a desirable level.

As the bitumen content increases from 8% to 9%, a general trend of reduced wet abrasion is observed (indicating enhanced abrasion resistance). However, this is accompanied by an increase in lateral displacement (indicating reduced stiffness and greater susceptibility to deformation). Therefore, the optimum point is where these two behaviors are balanced.

A residual bitumen content of 8.5% was identified as the most balanced and effective for mixtures modified with AgNPs or foundry waste filler. In contrast, for mixtures without nanomaterials or containing only natural filler, 8% residual bitumen provided better stability. The use of 9% bitumen is recommended only when higher workability is needed or in colder climates requiring softer mixes.

### Mixing time test

The mixing time test serves as a fundamental operational assessment for evaluating the workability and mixing behavior of SS mixtures. Its main objective is to determine the time required to achieve complete homogeneity among the bitumen emulsion, filler, aggregate, and other additives (such as nanomaterials) under standardized conditions. According to ISSA A143 guidelines, the minimum acceptable mixing time for field applications is 180 s.

In this study, mixing times were measured for various mixtures with different proportions of waste filler (ranging from 0% to 100%) and with or without the presence of AgNPs. The results of this test are summarized in Table 7.

The findings show that the WFP0 samples (natural filler only) and NWFP0 samples (0% waste filler + 10% nano) recorded mixing times ranging from 182 to 185 s—all exceeding the minimum required threshold. This indicates that the presence of nanoparticles alone does not negatively impact the mixing capability. Furthermore, as the amount of waste fillers increased from 25% to 100% in the NWFP25 to NWFP100 samples, a slight decrease in mixing time was observed, with most values remaining between 181 and 185 s. Specifically, for the NWFP50 sample, the mixing time ranged between 183 and 184 s, while NWFP100 showed the shortest observed time of 181 s.

The simultaneous presence of AgNPs (10% by weight of total filler) and foundry waste filler did not reduce the mixing time below the standard threshold. In fact, in most cases, the mixing time stabilized within the 183–185 s range, suggesting good compatibility of the nano and waste filler components with the mixing environment. All tested combinations met the ISSA minimum requirements for mixing time. The use of nanomaterials and waste fillers not only did not hinder the mixing process but also contributed to stable and uniform blending behavior. These results confirm the high practical feasibility of the modified mixtures in terms of processing time under field conditions.

**Table 7:** Mixing time test results for SS mixtures.

Mixture type	Waste filler replacement (%)	Nano content (%)	Residual Bitumen (%)	Water content (%)	Measured mixing time (s)
WFP0	Natural filler only	–	8.0	6.0	183
WFP0	Natural filler only	–	8.5	6.0	185
WFP0	Natural filler only	–	9.0	6.5	185
NWFP0	Natural filler only	10	8.0	6.0	182
NWFP0	Natural filler only	10	8.5	6.0	183
NWFP0	Natural filler only	10	9.0	6.5	184
NWFP25	25% waste filler	10	8.0	7.0	182
NWFP25	25% waste filler	10	8.5	7.0	183
NWFP25	25% waste filler	10	9.0	7.5	181
NWFP50	50% waste filler	10	8.0	8.0	184
NWFP50	50% waste filler	10	8.5	8.0	184
NWFP50	50% waste filler	10	9.0	8.0	183
NWFP75	75% waste filler	10	8.0	8.5	182
NWFP75	75% waste filler	10	8.5	9.0	181
NWFP75	75% waste filler	10	9.0	9.0	185
NWFP100	100% waste filler	10	8.0	9.0	181
NWFP100	100% waste filler	10	8.5	9.5	184
NWFP100	100% waste filler	10	9.0	9.5	183

### Wet cohesion test (WCT)

**Fig. 6:**
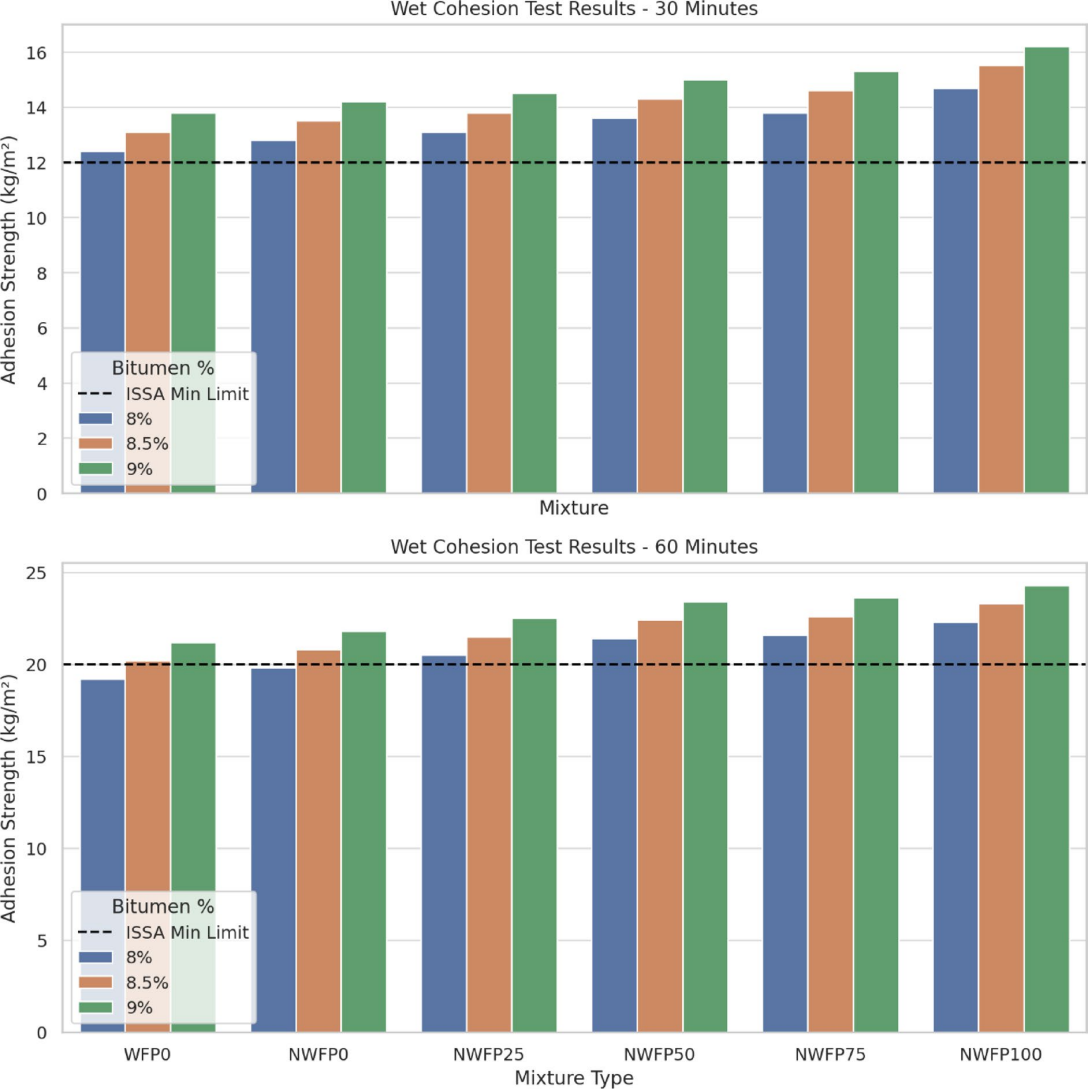
Measured torque values (kg·cm) at 30 and 60 min.

As shown in Fig. 6, the results of the wet cohesion test (WCT) were evaluated for all mixtures at two intervals: 30 and 60 min. This test was conducted in accordance with ISSA TB 105, with the aim of assessing the traffic-opening capability and initial strength of the mastics under wet conditions.

All mixtures successfully met the minimum wet cohesion values specified by the ISSA standard. Specifically, at both 30 and 60 min, all formulations exceeded the required thresholds, indicating that traffic reopening could be achieved within standard time frames. A consistent trend was observed: increasing the residual bitumen content from 8% to 9% enhanced wet cohesion in all mixtures. Among the nano-modified formulations, NWFP75 and NWFP100 exhibited the strongest performance, confirming the beneficial effect of combining waste filler with AgNPs. Overall, the results demonstrate that incorporating higher levels of waste filler together with AgNPs significantly improves cohesion, particularly at higher bitumen contents, thereby enhancing durability.

The enhanced cohesion observed in mixtures containing both foundry waste filler and AgNPs can be explained by the increased surface area and reactivity of nanoparticles, which promote stronger chemical interactions within the mastic. Moreover, the angular texture of waste filler particles contributes to better interlocking with the binder, delaying stripping and ensuring adequate strength for early traffic opening.

### Wet track abrasion test (WTAT)

**Fig. 7:**
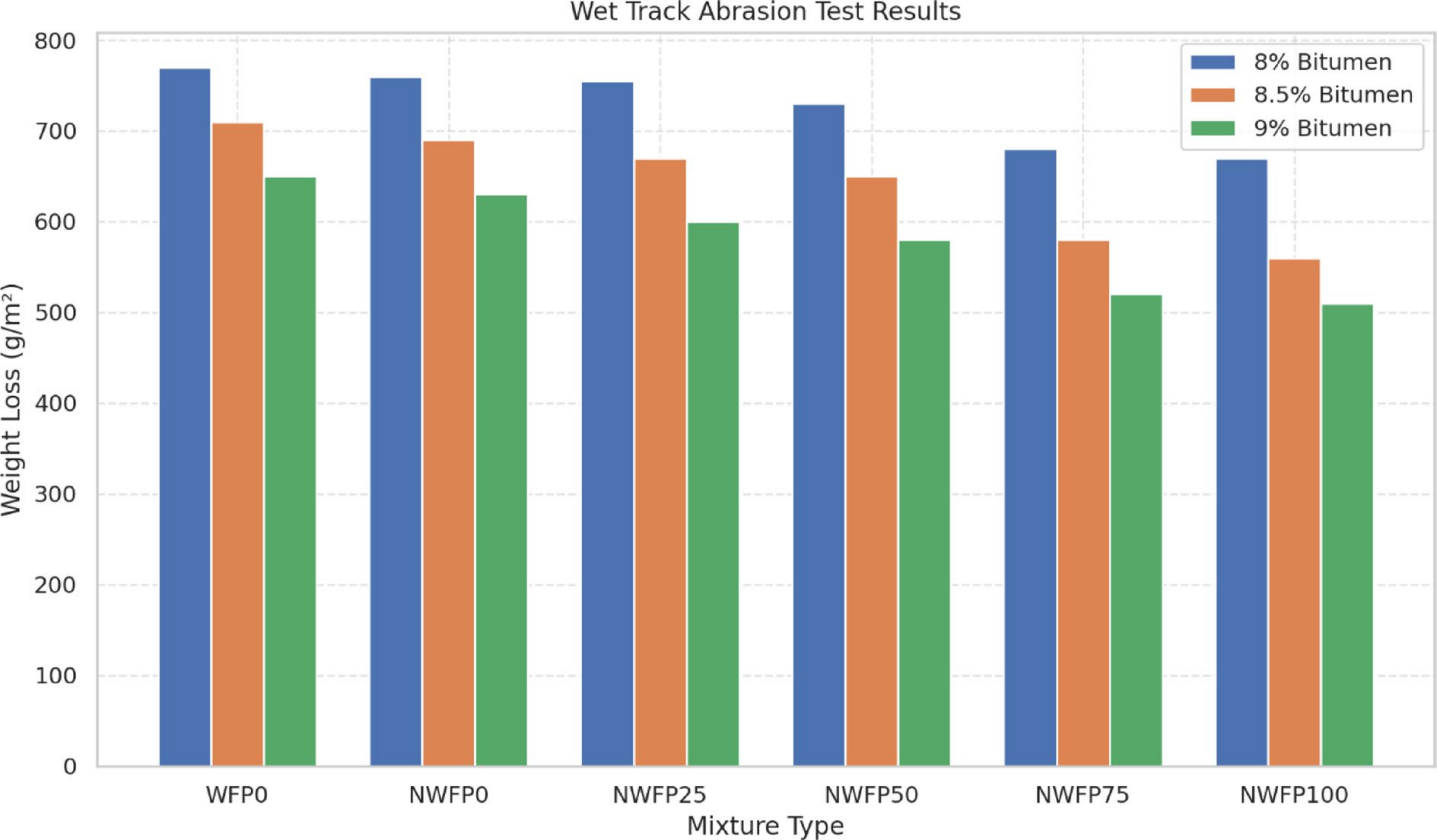
Weight loss due to abrasion for the studied SS mixtures.

This test was conducted to evaluate the abrasion resistance of modified SS mixtures under wet loading conditions over a one-hour period. As illustrated in Fig. 7, increasing the residual bitumen content from 8% to 9% consistently reduced abrasion-related weight loss across all mixtures. This improvement is attributed to enhanced cohesion of the bitumen matrix and reduced water permeability. The nano-modified mixtures, especially NWFP100, exhibited the lowest abrasion losses, confirming the positive effect of combining waste filler with AgNPs. While NWFP75 performed well at lower bitumen levels, NWFP100 showed the best overall resistance at 9%. All mixtures complied with ISSA specifications, and the results highlight that the proper selection of filler replacement ratio and bitumen content can significantly improve surface durability under wet conditions.

The NWFP samples—especially NWFP100, which contains 100% waste filler and 10% AgNPs—exhibited superior performance in minimizing weight loss due to abrasion. For example, the NWFP100 mixture with 9% residual bitumen recorded a weight loss of approximately 510 g/m², which was significantly lower (by about 32%) than that of the control sample (WFP0) under the same conditions.

At 8% residual bitumen, the weight loss for samples WFP0 through NWFP100 was 740, 703, 699, 645, and 620 g/m², respectively. At 8.5%, these values were 690, 660, 630, 590, and 550 g/m². At 9%, further improvement was observed with the weight loss values of 660, 630, 590, 520, and 510 g/m², respectively. These results clearly demonstrate that increasing the residual bitumen not only enhances abrasion resistance but also shifts the optimal filler content. While NWFP75 showed the best performance at 8% and 8.5% bitumen levels, NWFP100 outperformed all mixtures at 9%.

All mixtures tested in this study complied with the ISSA standard for maximum allowable weight loss. The best abrasion resistance was achieved with the NWFP100 mixture at 9% residual bitumen. Therefore, for optimizing abrasion performance, it is recommended to adjust the bitumen content based on the type and percentage of filler and nanoparticles used. These findings indicate that the proper selection of bitumen percentage and modified filler type can significantly enhance the surface durability of protective asphalt under wet conditions.

The superior abrasion resistance in NWFP mixtures, particularly NWFP100, reflects the synergistic role of siliceous waste filler and silver nanoparticles. While the filler enhances stiffness and reduces permeability, AgNPs improve binder cohesion by forming a more stable network within the emulsion matrix. This dual mechanism explains why higher filler replacement combined with nanoparticles reduces aggregate loss under wet abrasive loading.

### Loaded wheel test

**Fig. 8:**
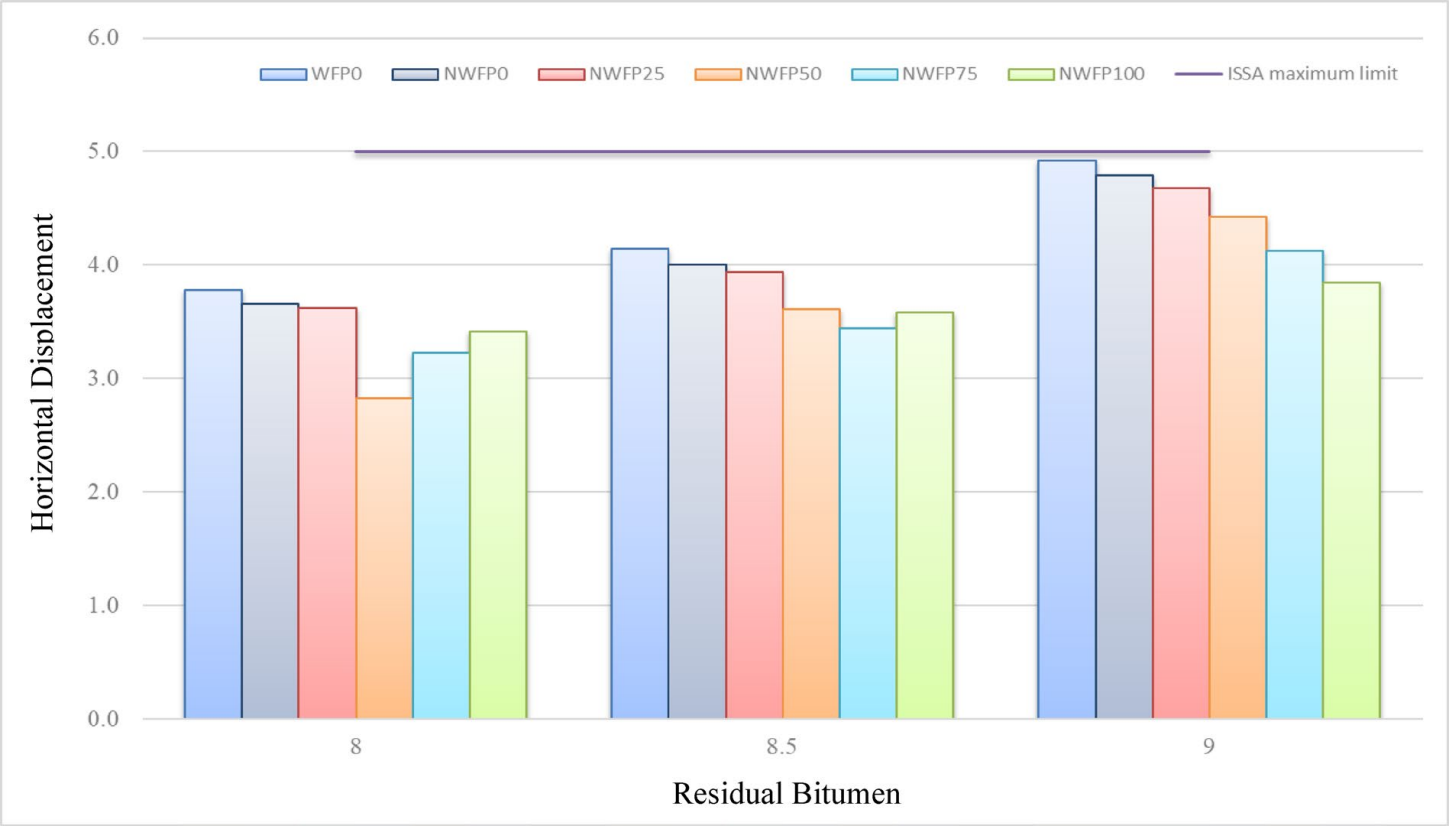
Horizontal displacement of different mixtures at residual Bitumen contents of 8% to 9%.

**Fig. 9:**
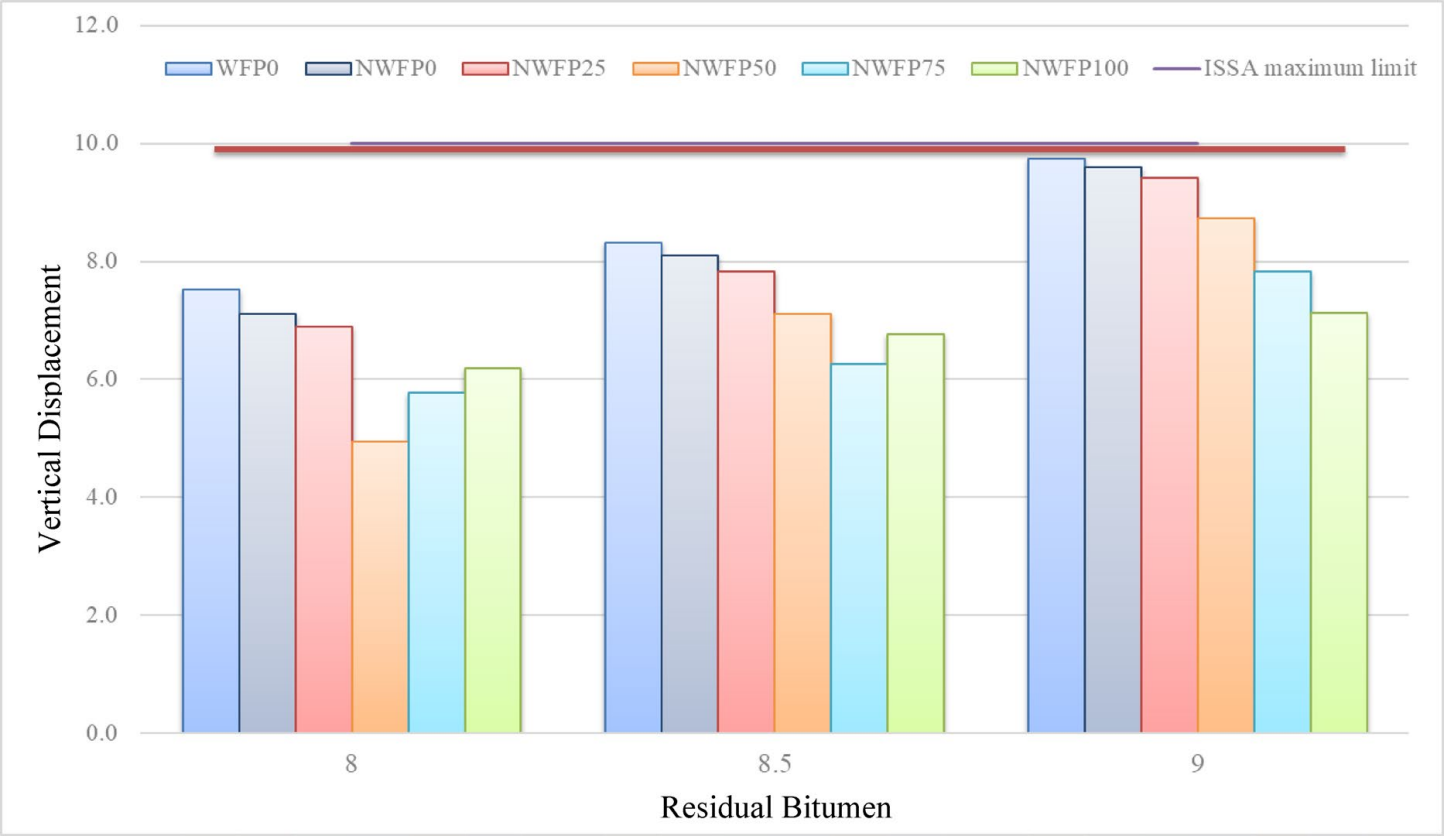
Vertical displacement of different mixtures at residual Bitumen contents of 8% to 9%.

Figures 8 and 9 present the horizontal and vertical displacement results for different mixtures. As residual bitumen increased from 8% to 9%, both horizontal and vertical displacements rose, reflecting greater softness of the bitumen matrix. However, nano-modified mixtures with higher waste filler contents exhibited noticeably reduced deformation compared to the control, especially at intermediate bitumen levels. Among all, NWFP50 at 8.5% residual bitumen consistently recorded the lowest displacements in both directions, providing the most favorable performance for rutting resistance.

As shown in Fig. 8, at a residual bitumen content of 8.5%, the horizontal displacement for the control sample (WFP0) was 4.2%, while for the NWFP0, NWFP25, NWFP50, NWFP75, and NWFP100 mixtures, the values were 4.1%, 3.9%, 3.5%, 3.7%, and 3.8%, respectively. This notable reduction highlights the significant role of AgNPs and foundry waste filler in controlling horizontal displacement under wheel loading conditions.

The NWFP50 mixture exhibited the lowest horizontal displacement, well below the 5% limit specified by ISSA, offering a considerable safety margin. According to Fig. 9, the vertical displacement values for the same mixtures at 8.5% residual bitumen were 8.1%, 7.2%, 6.4%, 6.8%, and 6.9%, respectively. The results indicate a substantial reduction in vertical displacement with increasing waste filler content. Again, the NWFP50 mixture showed superior performance in minimizing vertical deformation.

In both horizontal and vertical displacement graphs, NWFP50 at 8.5% residual bitumen outperformed all other mixtures. This formulation achieved the lowest displacements in both directions while fully complying with ISSA specifications, making it the most favorable mixture in terms of rutting resistance under wet loading conditions. In conclusion, the NWFP50 mixture with 8.5% residual bitumen demonstrated the best overall performance for minimizing both horizontal and vertical displacements, outperforming both the control and other modified mixtures.

The lower horizontal and vertical displacements recorded in nano-modified mixtures can be attributed to the reinforcing effect of silver nanoparticles, which improve mastic stiffness and reduce susceptibility to deformation. Additionally, waste filler provides a rigid skeletal framework, counteracting the softening effect of higher bitumen contents. This balance results in enhanced rutting resistance, especially in NWFP50 at 8.5% residual bitumen.

### Loaded wheel and sand adhesion test (LWSAT)

**Fig. 10:**
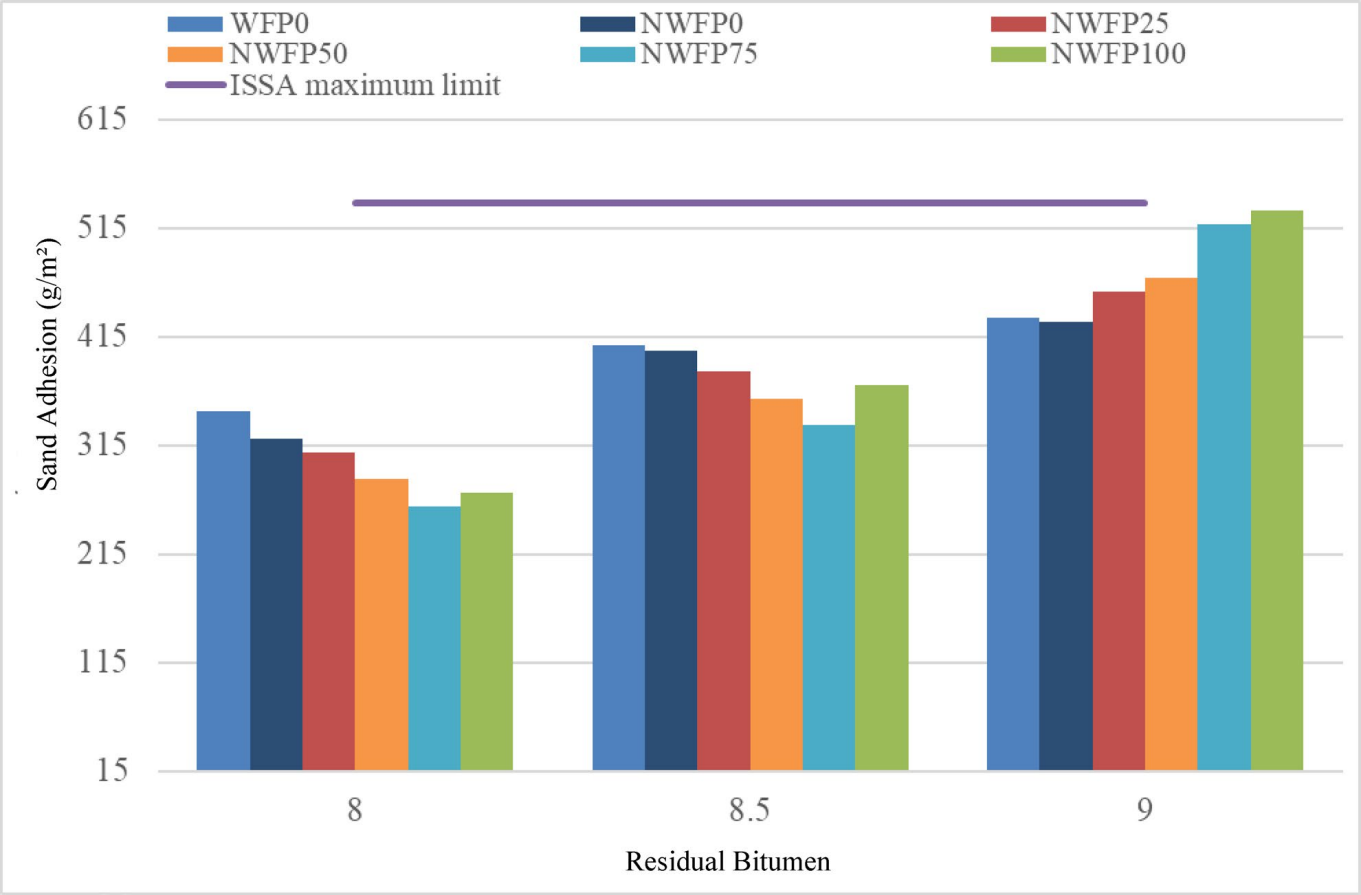
Sand adhesion results of different mixtures.

In this study, sand adhesion is denoted as MSA (Mass of Sand Adhesion), and this abbreviation is used consistently throughout the manuscript. Figure 10 displays the sand adhesion test results. All mixtures remained below the ISSA critical limit, confirming adequate bleeding resistance. As bitumen content increased, sand adhesion values also rose, but the modified mixtures with waste filler and AgNPs—particularly NWFP75 and NWFP100—consistently exhibited lower adhesion than the control. This indicates superior resistance to bleeding. The overall trend suggests that optimizing both the filler replacement ratio and residual bitumen content is key to controlling surface bleeding, with NWFP75 at 8.5% offering the most balanced performance.

The residual bitumen content had a significant impact on improving sand adhesion. As the bitumen content increased from 8% to 9%, sand adhesion values (an indicator of surface bleeding) also increased. This trend was observed in all mixtures and was particularly notable in formulations modified with both foundry waste filler and AgNPs (e.g., NWFP75 and NWFP100). Nevertheless, all measured values remained below the critical limit, indicating successful control of bitumen bleeding in these mixtures.

At 8% bitumen, the lowest sand adhesion was recorded for sample WFP0 with 340 g/m², while the highest was for NWFP100 with about 270 g/m² (approximately 20% lower than WFP0), indicating superior bleeding control in the modified sample. At 8.5%, NWFP50 and NWFP75 showed further reductions in sand adhesion compared to the control. For instance, NWFP50 recorded 360 g/m², which was 12.1% lower than WFP0 (410 g/m²). At 9%, the best performance in terms of minimum sand adhesion was observed in NWFP100 with 525 g/m², close to the critical limit. Compared to WFP0, NWFP50, NWFP75, and NWFP100 showed improved performance by 10.7%, 17.9%, and 21.4%, respectively.

The results of the loaded wheel sand adhesion test indicate that increasing the proportion of waste filler, in combination with higher bitumen content (especially at 8.5%), can significantly enhance anti-bleeding performance without exceeding critical limits. Among all mixtures, NWFP75 at 8.5% residual bitumen showed the best bleeding control and was identified as the optimal formulation based on sand adhesion performance under test conditions^22^. For practical use, it is recommended to simultaneously optimize both the bitumen content and filler replacement ratio to achieve the best results.

The improved resistance to bleeding observed in NWFP mixtures arises from the filler’s ability to absorb excess binder while nanoparticles enhance cohesion at the mastic level. Together, these mechanisms limit bitumen migration to the surface, thereby maintaining coating uniformity and reducing the risk of flushing. This explains why NWFP75 and NWFP100 consistently performed better than the control mix.

### Statistical analysis and modeling

The objective of this section is to utilize the parameters calculated in the previous sections to develop predictive models for sand adhesion (MSA) and weight loss due to abrasion (WLL) in SS samples, using two approaches: multivariate regression and genetic programming (GP).

Ultimately, by applying a dual-objective optimization process to these models, the optimal replacement percentage of foundry waste filler will be determined to enhance SS performance. In this study, MSA (Mass of Sand Adhesion) and WLL (Weight Loss due to Loading) were selected as the dependent variables. The independent input variables used in the predictive modeling include:

- Waste Filler Replacement (WFR).
- Silver Nanoparticles Content (AgNPs%).
- Bitumen Residue Percentage (BR%).
- Vertical Displacement (VD).
- Lateral Displacement (LD).
- Adhesion at 30 min (Adh30).
- Adhesion at 60 min (Adh60).

#### Multivariate regression model

##### Sand adhesion prediction model

Table 8 presents the coefficients of the multivariate regression model along with the statistical significance tests for each coefficient. These coefficients, listed under the “Unstandardized Coefficients” column, represent the influence of each variable in the linear regression model developed to predict sand adhesion based on seven independent variables.

The analysis showed that the direction and magnitude of influence varied among the independent variables. Waste filler replacement (WFR) and silver nanoparticle content (AgNPs%) had negative coefficients, indicating that higher values of these inputs reduced sand adhesion. In contrast, the bitumen residue percentage (BR%) had a positive coefficient, suggesting that an increase in residual bitumen enhances sand adhesion.

Vertical and lateral displacements had relatively weak effects and were statistically insignificant. Adhesion at 30 min showed a strong positive influence, confirming that initial cohesion plays a key role in final sand adhesion. Adhesion at 60 min had a limited but still positive effect.

Overall, the model demonstrated a very good fit, indicating possible interactions among variables or underlying nonlinear effects in the system.

**Table 8:** Coefficients of the proposed regression model.

Model variable	Unstandardized coefficient (B)	Standard error	t−value	*p*−value
Constant	−1222.184	280.6	−4.36	0.001
Waste filler replacement (%)	−1.512	0.836	−1.81	0.004
AgNPs content (%)	−3.804	3.009	−1.26	0.02
Residual Bitumen content (%)	840.932	659.1	1.27	0.03
Vertical displacement (%)	40.373	450.6	0.09	0.97
Horizontal displacement (%)	371.426	1931.0	0.16	0.16
Adhesion at 30 min (kg·cm)	710.5	704.8	1.0	0.000
Adhesion at 60 min (kg·cm)	−836.0	672.6	−1.25	0.03

Based on the coefficients provided, the regression model developed in this study can be expressed as:

5 $$\begin{aligned} {\text{MSA}} & {\text{=-1222}}{\text{.84-0}}{\text{.512WFR-3}}{\text{.804AgNPs}}\,{\text{+}}\,{\text{84}}{\text{.093BR}}\, \\ & {\text{+}}\,{\text{7}}{\text{.523VD}}\,{\text{+}}\,{\text{32}}{\text{.847LD}}\,{\text{+}}\,{\text{71}}{\text{.05Adh30-8}}{\text{.356Adh60}} \\ \end{aligned}$$

Where:

WFR is the Waste Filler Replacement (%), AgNPs% is the AgNPs Content (%),

BR% is the Residual Bitumen Content, VD is the Vertical Displacement (%), LD is the Lateral Displacement (%), Adh30 is the Adhesion at 30 min (kg·cm), and Adh60 is the Adhesion at 60 min (kg·cm). Most importantly, MSA represents the predicted Mass of Sand Retention.

A summary of the developed model is presented in Table 9. According to the table, the coefficient of determination (R²) for the proposed model is 0.887, while the multiple correlation coefficient (R), which is the square root of R², is 0.942.

The use of the adjusted R² is important in multivariate regression, as the inclusion of even weakly correlated independent variables can artificially inflate the R² value. The adjusted R² accounts for the number of predictors and the sample size, making it a more reliable metric—particularly when comparing different models. Essentially, it compensates for unequal numbers of variables across models. Additionally, the Root Mean Square Error (RMSE) of the model was calculated as 26.28.

**Table 9:** Summary of regression model results using multivariate regression.

Model metric	Result
Multiple correlation coefficient (R)	0.942
Coefficient of determination (R^2^)	0.887
Root mean square error (RMSE)	26.28

**Fig. 11:**
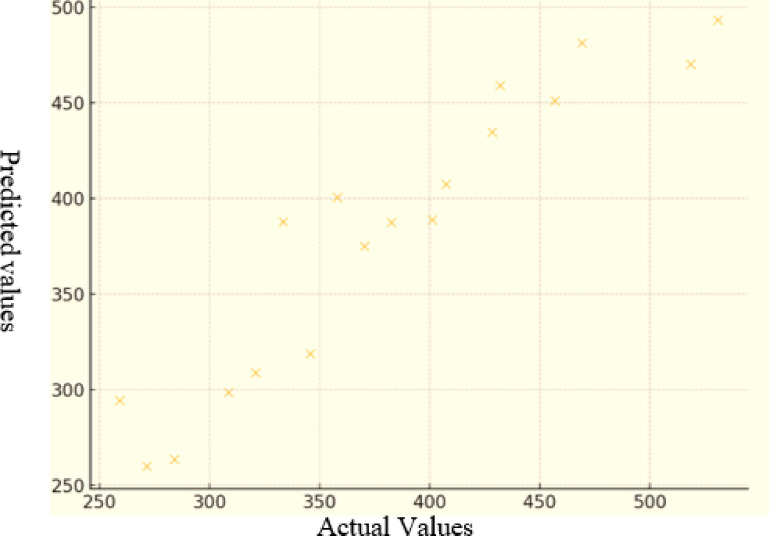
Comparison of actual and predicted values of the MSA model.

Based on the regression model presented in Eq. (5), the observed values, predicted values (calculated using the model), and the residuals (differences between observed and predicted values) were determined. As illustrated in Fig. 11, a comparison was made between the predicted values generated by the model and the actual measured data.

**Fig. 12:**
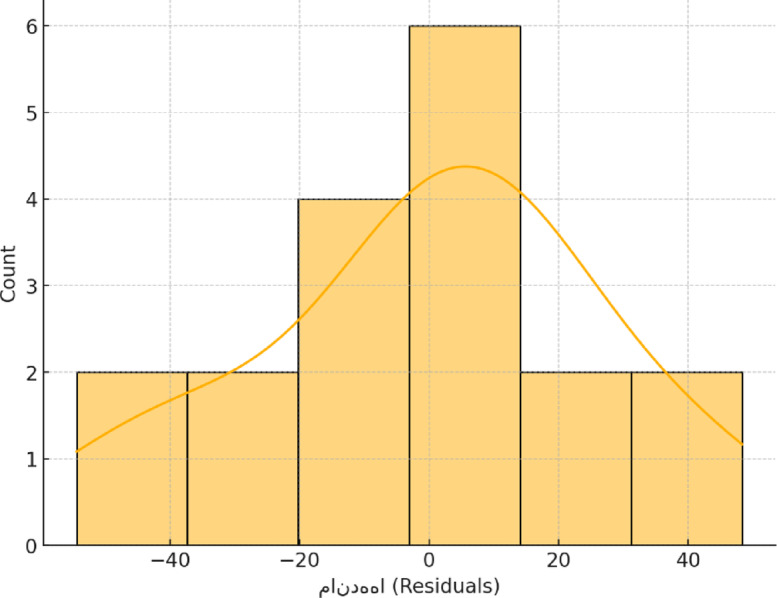
Normal distribution of residuals in the MSA model.

A key assumption underlying the application of multivariate regression is that the residuals are normally distributed—an assumption that becomes especially critical when working with small sample sizes. Figure 12 depicts the residual distribution, which appears to closely align with the characteristics of a normal distribution.

##### Weight loss perdiction

Table 10 presents the coefficients of the multivariate regression model, along with the significance tests for each coefficient. The regression coefficients are listed under the “Unstandardized Coefficients” column. A linear multivariate regression model was developed to estimate the Weight Loss due to Loading (WLL) based on seven independent variables, including:

- Waste filler replacement percentage.
- Silver nanoparticle content.
- Residual bitumen percentage.
- Vertical displacement.
- Lateral displacement.
- Adhesion at 30 min.
- Adhesion at 60 min.

Analysis of the regression coefficients showed that the residual bitumen content had a negative and statistically significant coefficient (*p* < 0.01). This means that increasing the residual bitumen content significantly reduces abrasion-induced weight loss. This finding is consistent with expectations, as increased binder adhesion helps prevent the dislodging of sand particles under abrasion forces.

Vertical displacement had a positive coefficient, and with approximately 90% confidence, it can be concluded that greater vertical deformation leads to increased weight loss. This is logical, as higher displacement is an indicator of structural weakness and increased deformability.

The waste filler replacement also had a negative coefficient, implying that increasing the amount of waste filler generally resulted in a reduction in abundance. The coefficient of silver nanoparticle content was slightly negative but very small, suggesting that its individual linear effect on abrasion may not be significant in isolation but could be influential in combination with other variables.

Other variables—Adhesion at 30 and 60 min and Lateral Displacement—were removed from the final model due to their statistical insignificance, to improve the model’s simplicity and accuracy.

The multivariate regression model demonstrated high precision and reliability in predicting abrasion-related weight loss based on mastic characteristics. The most critical findings were the inverse effect of residual bitumen content and the positive effect of vertical displacement on weight loss. These results can serve as a foundation for optimizing more abrasion-resistant asphalt mixtures under dynamic loading conditions.

**Table 10:** Coefficients of the proposed regression model.

Model variable	Unstandardized coefficient (B)	Standard error	t−value	*p*−value
Constant	3216.229	218.56	14.71	0.000
Waste filler replacement (%)	−0.79	0.48	−1.61	0.04
AgNPs content (%)	−0.10	2.34	−0.04	0.04
Residual Bitumen content (%)	−244.433	64.11	−3.79	0.003
Vertical displacement (%)	130.26	60.52	1.88	0.05
Horizontal displacement (%)	−285.29	151.00	−1.88	0.08

Based on the coefficients provided, the multivariate regression model developed in this study can be expressed as:

6 $${\text{WLL}}\,=\,{\text{3216}}.{\text{29 }} - 0.{\text{79WFR}} - 0.{\text{1AgNPs}} - {\text{243}}.{\text{43BR}}\,+\,{\text{13}}0.{\text{76VD}} - {\text{285}}.{\text{29LD}}$$

Where:

- WFR = Waste Filler Replacement (%).
- AgNPs = Silver Nanoparticles Content (%).
- BR = Bitumen Residue Percentage (%).
- VD = Vertical Displacement (%).
- LD = Lateral Displacement (%).
- WLL = Weight Loss due to Loading (g/m²).

A summary of the model performance is presented in Table 11. According to the table, the coefficient of determination (R²) for the proposed model is 0.978, and its square root—the multiple correlation coefficient (R)—is 0.989.

The use of the adjusted R² is important in multivariate regression, as adding even weakly correlated independent variables can artificially inflate the R² value. The adjusted R² accounts for both the number of predictors and the sample size, making it a more reliable measure. It is typically used for comparing different models, as it normalizes for the unequal number of variables across model structures.

Additionally, the Root Mean Square Error (RMSE) for this model was calculated to be 20.50, indicating a high degree of predictive accuracy.

**Table 11:** Summary of results for the MVR prediction model.

Model metric	Result
Multiple correlation coefficient (R)	0.898
Coefficient of determination (R^2^)	0.978
Root mean square error (RMSE)	20.51

**Fig. 13:**
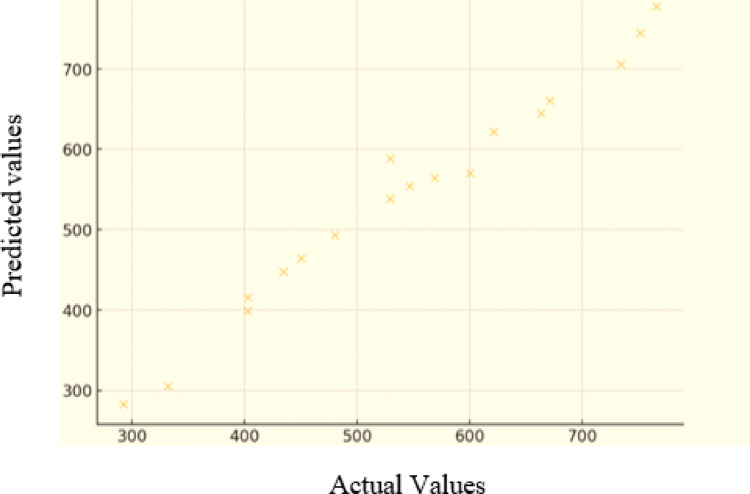
Comparison of actual and predicted values of the WLL model.

**Fig. 14:**
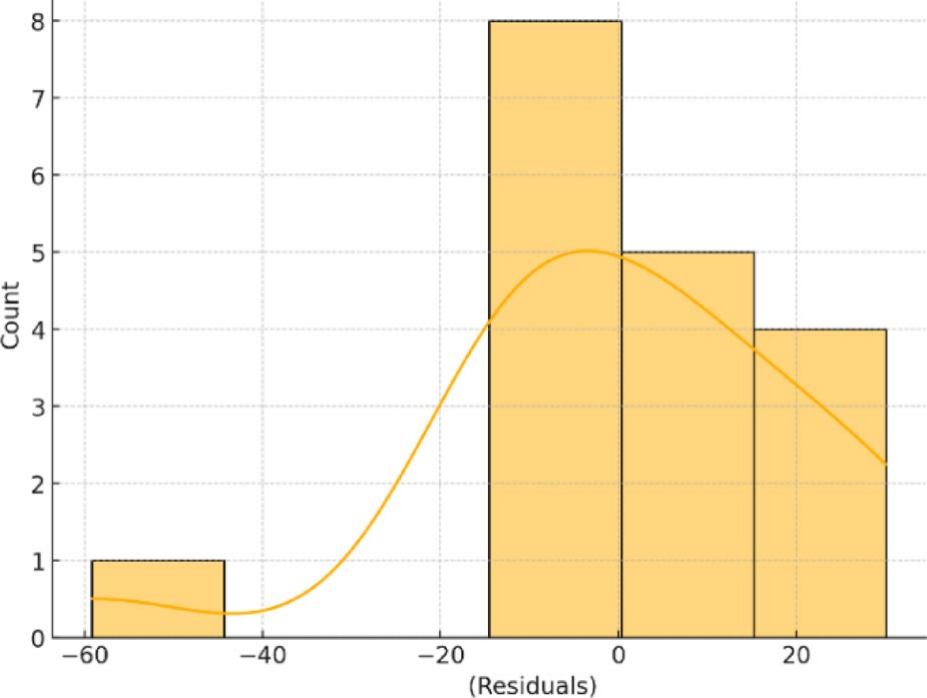
Normal distribution of residuals in the WLL model.

Using the regression model outlined in Eq. (6), the observed values, model-predicted values, and residuals (defined as the difference between observed and predicted values) were computed. Figure 13 presents a comparison between the predicted and actual measured data, illustrating the model’s predictive performance. A fundamental assumption in multivariate regression is that the residuals are normally distributed—an aspect that becomes particularly crucial when dealing with small sample sizes. As shown in Fig. 14, the distribution of residuals demonstrates a strong alignment with a normal distribution pattern, supporting the validity of this assumption.

#### Genetic programming (GP)

##### Sand adhesion prediction model

The results of the sand adhesion prediction model developed using the Genetic Programming (GP) method are presented in Eq. (7). A summary of the model’s performance is shown in Table 12, which indicates a coefficient of determination (R²) of 0.994 for this study. Additionally, the multiple correlation coefficient (R) is 0.996, and the Root Mean Square Error (RMSE) is 3.31.

**Fig. 15:**
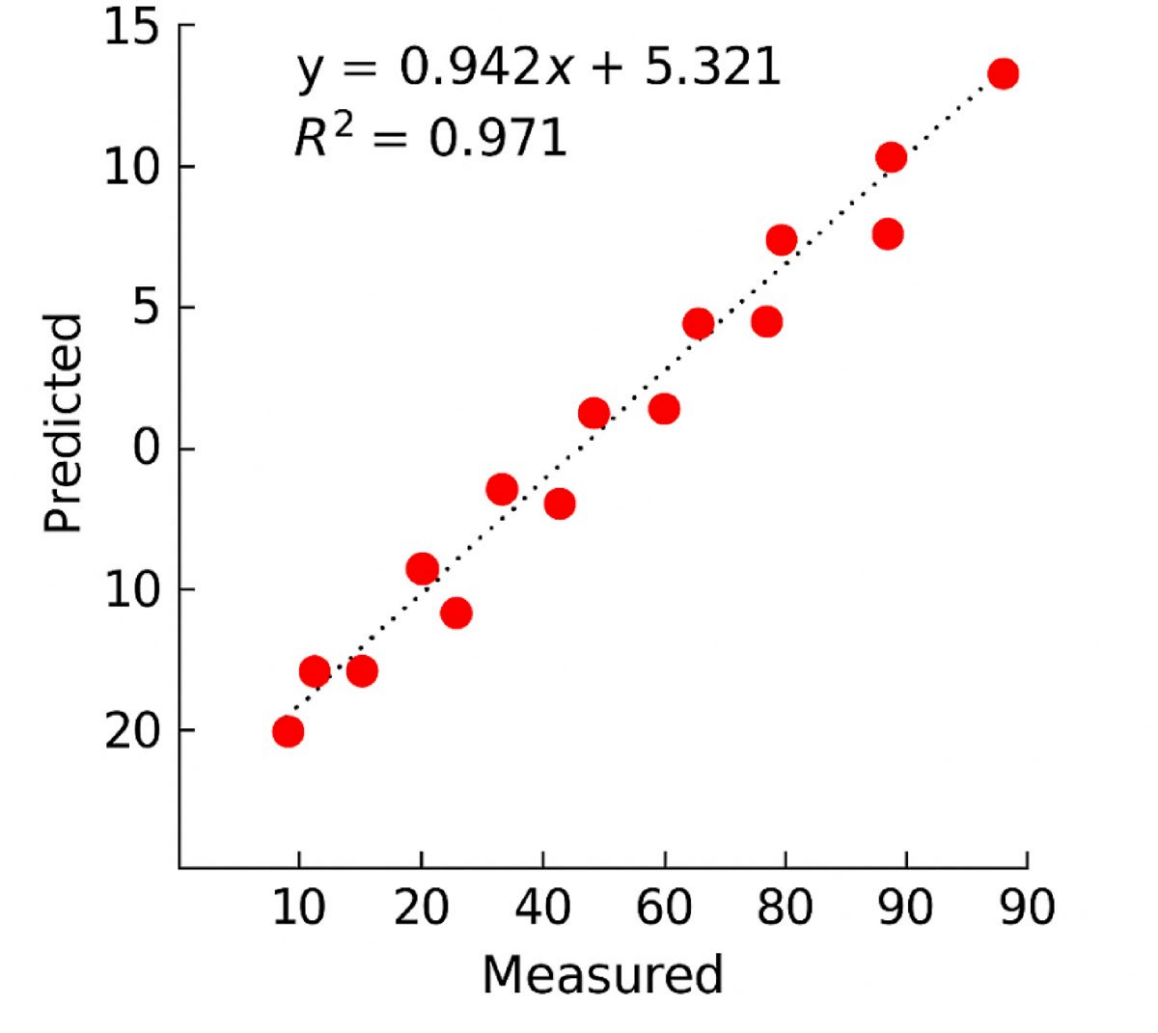
Comparison of actual and predicted sand adhesion values using the GP model.

Moreover, in Fig. 15, the predicted values obtained from the GP model are compared with the experimental values. As illustrated, the predicted values closely match the actual laboratory data, demonstrating the model’s high accuracy and reliability in capturing the sand adhesion behavior.

7 $$\begin{aligned} {\text{MSA}}\, & =\,{\text{Log }}({\text{cos }}\left( {{\text{WFR}}} \right)\,+\,{\text{sin }}\left( {{\text{AgNPs}}} \right))\,+\,{\text{23}}.0{\text{14B}}{{\text{R}}^{\text{2}}} - {\text{52}}.{\text{42V}}{{\text{D}}^{\text{3}}}\, \\ & +\,{\text{cos }}\left( {{\text{ex}}{{\text{p}}^{{\text{5}}.{\text{27LD}}}}} \right)\,+\,{\text{sinAdh3}}0 \times {\text{cos}}\left( {{\text{ex}}{{\text{p}}^{{\text{1}}.{\text{33Adh6}}0}}} \right) \\ \end{aligned}$$

Where:

- WFR = Waste Filler Replacement (%).
- AgNPs = Silver Nanoparticles Content (%).
- BR% = Bitumen Residue Percentage.
- VD = Vertical Displacement (%).
- LD = Lateral Displacement (%).
- Adh30 = Adhesion at 30 min.
- Adh60 = Adhesion at 60 min.
- and most importantly, MSA represents the predicted value of Mass of Sand Adhesion.

**Table 12:** Summary of results for the GP-based prediction model.

Model metric	Result
Multiple correlation coefficient (R)	0.996
Coefficient of determination (R^2^)	0.994
Root mean square error (RMSE)	3.31

##### Weight loss perdiction

The results of the sand adhesion prediction model based on the Genetic Programming (GP) method are presented in Eq. (8). A summary of the model’s performance is provided in Table 13, indicating that the coefficient of determination (R²) achieved in this study is 0.994. Additionally, the multiple correlation coefficient (R) is 0.996, and the Root Mean Square Error (RMSE) is 3.31.

**Fig. 16:**
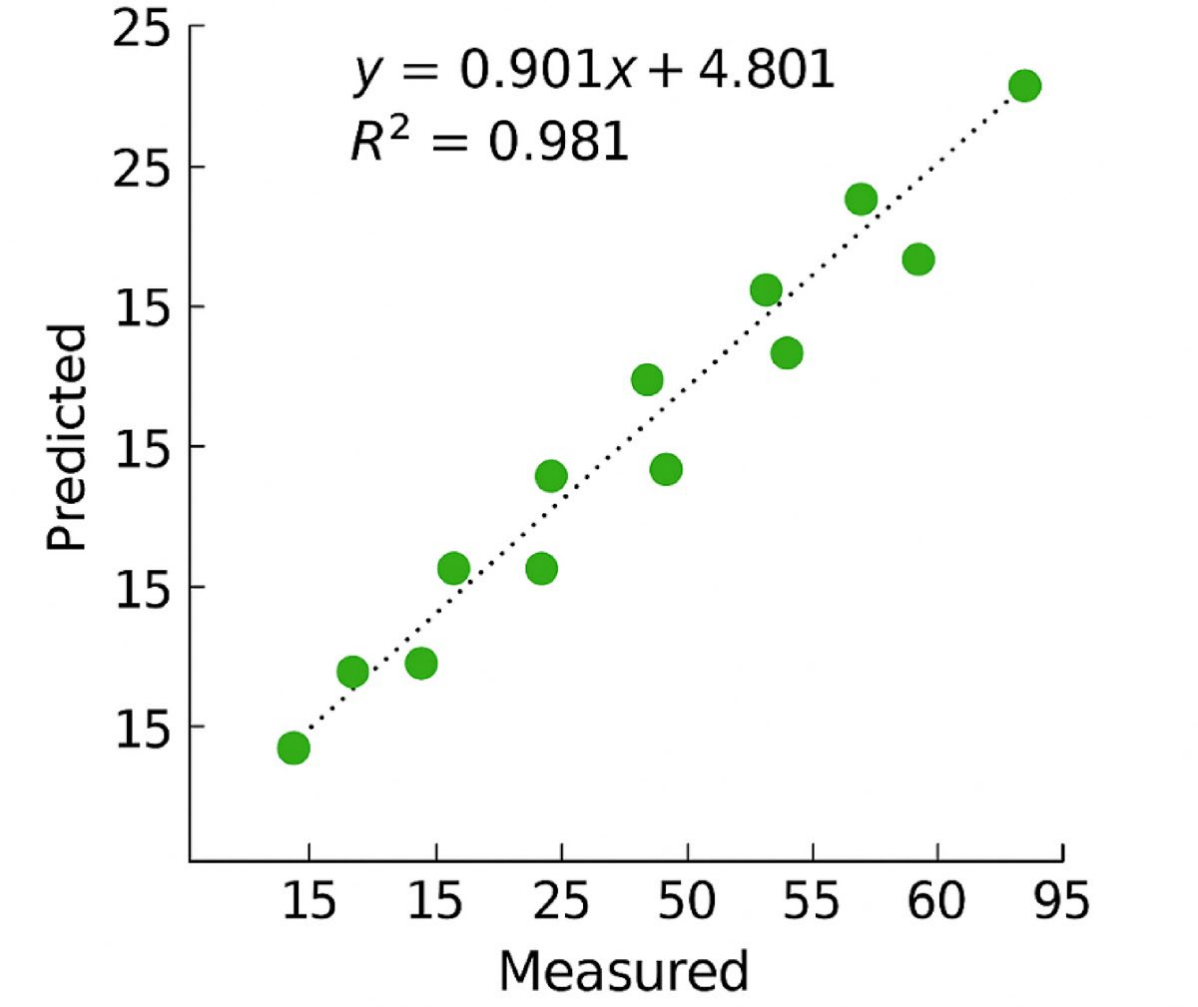
Comparison of actual and predicted sand adhesion values using the GP model.

Furthermore, Fig. 16 compares the predicted values obtained from the GP model with the actual laboratory results. As shown, the predicted values accurately reflect the experimental data, demonstrating the model’s strong predictive capability and reliability in estimating sand adhesion behavior.

8 $$\begin{aligned} {\text{WFR}}\, & =\,{\text{87}}.{\text{28}}{\left( {{\text{WFR}}} \right)^{\text{3}}}+{\text{ 2}}.{\text{564ex}}{{\text{p}}^{{\text{1}}.{\text{462}}({\text{AgNPs}}))}}--{\text{63}}.{\text{12cos }}({\text{BR}} \times {\text{VD}})\, \\ & +\,{\text{log}}0.{\text{55LD}}\,+\,{\text{5}}.{\text{33Adh3}}{0^{\text{2}}} - {\text{2}}.{\text{44 cos}}\left( {{\text{3}}.{\text{31Adh6}}0} \right) \\ \end{aligned}$$

Where:

- WFR = Waste Filler Replacement (%).
- AgNPs = Silver Nanoparticles Content (%).
- BR% = Bitumen Residue Percentage.
- VD = Vertical Displacement (%).
- LD = Lateral Displacement (%).
- and most importantly, WLL represents the Weight Loss due to Loading.

**Table 13:** Summary of results for the GP-based prediction model.

Model metric	Result
Multiple correlation coefficient (R)	0.994
Coefficient of determination (R^2^)	0.990
Root mean square error (RMSE)	1.74

#### Optimization of additive dosages

Multi-objective optimization enables designers to select a solution from the Pareto front based on specific project requirements. In such problems, multiple objective functions are defined that one aims to minimize or maximize simultaneously^23^. Often, these objectives are conflicting, meaning that improving one may lead to the deterioration of another. Therefore, a single global optimum that satisfies all objectives does not exist. Instead, a set of optimal solutions, known as the Pareto front, is identified. This fundamental difference distinguishes multi-objective problems from single-objective ones^24^. These solutions, which include optimal trade-offs, help guide the design process more transparently and effectively.

The objective of this section is to optimize the waste filler replacement percentage in SS mixtures containing 10% AgNPs. The waste filler was used in varying proportions ranging from 25% to 100%. In the case that decision-makers opt for the application of protective SS asphalt, this optimization would contribute to the economic efficiency of the project^25^.

**Fig. 17:**
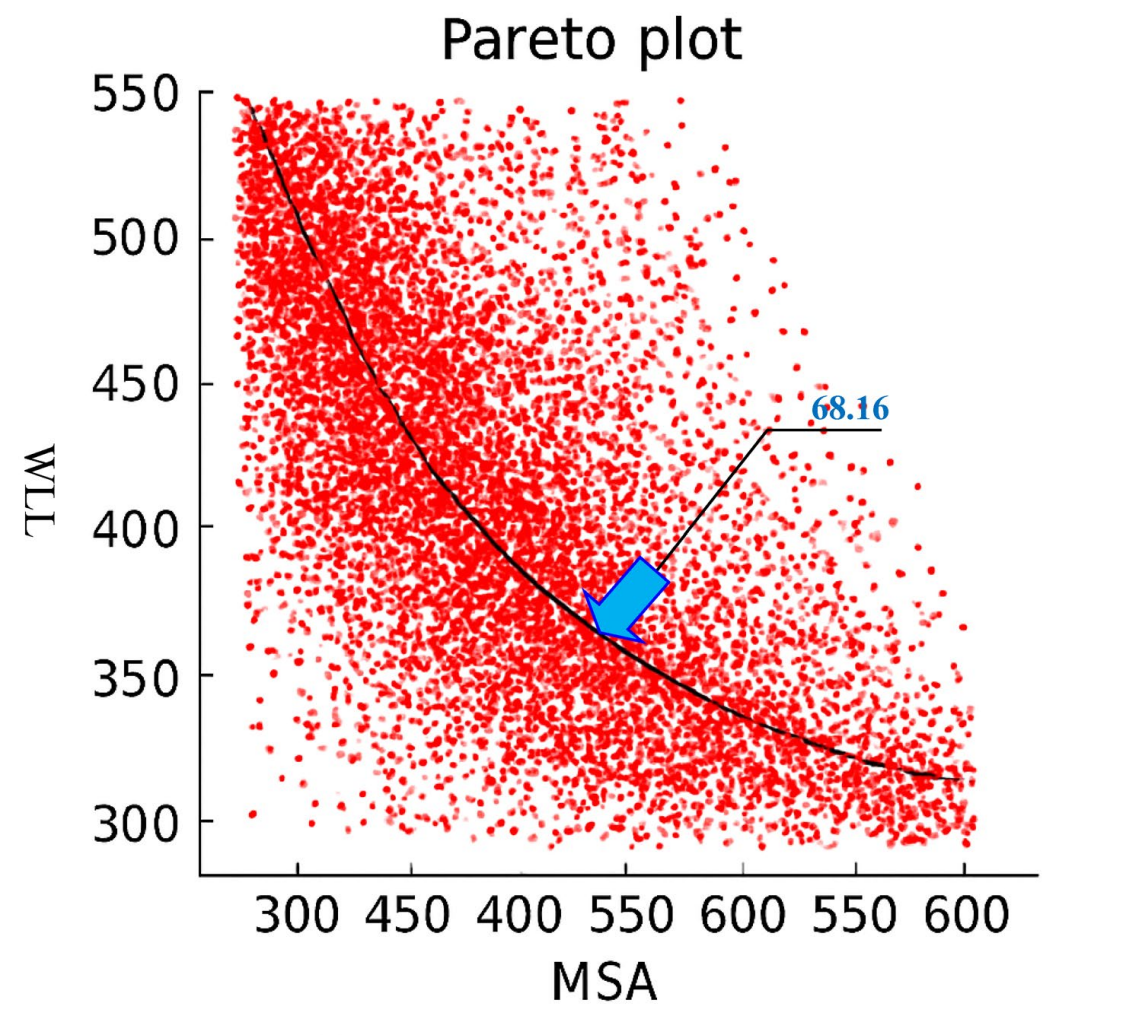
Pareto chart for the multi-objective optimization of MSA and WLL.

Before carrying out the optimization process, it is necessary to compare the predictive models to determine which one yields the most accurate results. Table 14 presents the performance of both multivariate regression and Genetic Programming (GP) models. Based on the comparison of determination coefficients (R²), RMSE values, and other key indicators, it is evident that the GP method provides superior accuracy for both target variables—MSA (Mass of Sand Adhesion) and WLL (Weight Loss due to Loading). Therefore, the GP models were selected for bi-objective optimization of MSA and WLL to determine the optimal waste filler content. The resulting Pareto front is shown in Fig. 17, where the point corresponding to 68.16% waste filler replacement is identified as the optimal design solution. At this point, both MSA and WLL achieve simultaneously optimal performance, striking a balance between durability and resistance to stripping.

**Table 14:** Results obtained from predictive models using different methods.

Modeling method	RMSE	*R*^2^ (Coefficient of determination)	*R* (Multiple correlation coefficient)
MSA model prediction			
Multivariate regression	26.28	0.887	0.942
GP (Genetic programming)	23.10	0.994	0.997
WLL model prediction			
Multivariate regression	20.51	0.978	0.989
GP (Genetic programming)	17.40	0.990	0.994

As summarized in Table 14, both MVR and GP provided high predictive accuracy; however, GP consistently outperformed MVR. This improvement is not only numerical (higher R², lower RMSE) but also methodological. MVR, being limited to linear combinations of predictors, is less effective in capturing nonlinearities and complex interaction effects among variables such as waste filler replacement, silver nanoparticle content, and residual bitumen. In contrast, GP leverages evolutionary search to model nonlinear relationships and higher-order interactions automatically, without requiring predefined functional forms. This ability allows GP to better reflect the intrinsic complexity of material behavior in SS mixtures, where synergistic effects between fillers, nanoparticles, and bitumen strongly influence performance outcomes^26^.

## Conclusion

This study addressed the critical challenge of enhancing the durability and sustainability of protective Slurry Seal (SS) asphalt mixtures by reusing foundry waste filler and incorporating AgNPs as performance enhancers. A comprehensive experimental program was designed, including mixing time, cohesion, abrasion, wheel tracking, and sand adhesion tests, complemented by advanced statistical modeling and multi-objective optimization. This integrated methodology enabled a holistic assessment of both material properties and predictive modeling approaches. Based on the evaluation of the laboratory test results, the principal findings of this study can be summarized as follows:

- WCT results indicated that increasing the residual bitumen content and incorporating AgNPs significantly enhanced initial adhesion, with NWFP75 showing the best performance.
- In the WTAT (Wet Track Abrasion Test), the use of foundry waste filler combined with a higher bitumen content (9%) led to a substantial reduction in abrasion-induced weight loss; NWFP100 showed up to 32% improvement compared to the control sample.
- The Loaded Wheel Test revealed that the NWFP50 mixture with 8.5% residual bitumen had the lowest horizontal and vertical displacement, making it the most stable formulation against rutting.
- All mixtures remained within the acceptable range of the sand adhesion test, with NWFP50 and NWFP75 demonstrating superior control over bitumen bleeding.
- Using multivariate regression analysis, two linear models were developed to predict Mass of Sand Adhesion (MSA) and Weight Loss due to Loading (WLL). The models achieved R² values of 0.887 and 0.978, respectively.
- The genetic programming (GP)-based prediction models provided higher accuracy, effectively capturing complex behaviors and interdependencies among the input variables.
- By applying GP models in conjunction with the NSGA-II multi-objective optimization algorithm, a Pareto front was generated to simultaneously optimize both MSA and WLL. The optimal design point was identified at 68.16% waste filler replacement, at which both objectives reached their best values. This composition successfully balances surface durability, bleeding resistance, workability, and mastic stability.

This study demonstrates that using foundry waste filler in combination with AgNPs not only presents a sustainable and cost-effective solution for repurposing industrial waste but, when properly designed, can significantly enhance the durability and performance of protective asphalt layers. Moreover, the application of advanced modeling and optimization techniques enables more accurate engineering decision-making for real-world project conditions. These findings can serve as a foundation for the development of technical guidelines, the advancement of innovative construction materials, and the implementation of sustainable protective asphalt solutions nationwide.

## Data Availability

The datasets used and/or analysed during the current study are available from the corresponding author on reasonable request.
